# TRPV Channels in Osteoarthritis: A Comprehensive Review

**DOI:** 10.3390/biom14030292

**Published:** 2024-02-29

**Authors:** Changshun Chen, Fei Yang, Rongjin Chen, Chenhui Yang, Hefang Xiao, Bin Geng, Yayi Xia

**Affiliations:** 1 Department of Orthopedics, Lanzhou University Second Hospital, Lanzhou 730030, China; chencs666@ynu.edu.cn (C.C.); 120220901491@lzu.edu.cn (F.Y.); 120220901411@lzu.edu.cn (R.C.); 120220901481@lzu.edu.cn (C.Y.); 120220901471@lzu.edu.cn (H.X.); ery_gengb@lzu.edu.cn (B.G.); 2 Orthopedic Clinical Medical Research Center and Intelligent Orthopedic Industry Technology Center of Gansu Province, Lanzhou 730030, China; 3 The Second School of Clinical Medical, Lanzhou University, Lanzhou 730030, China; 4 Department of Orthopedics and Trauma Surgery, Affiliated Hospital of Yunnan University, Kunming 650032, China

**Keywords:** osteoarthritis, TRPV channels, inflammation, mechanotransduction, therapeutic strategies

## Abstract

Osteoarthritis (OA) is a debilitating joint disorder that affects millions of people worldwide. Despite its prevalence, our understanding of the underlying mechanisms remains incomplete. In recent years, transient receptor potential vanilloid (TRPV) channels have emerged as key players in OA pathogenesis. This review provides an in-depth exploration of the role of the TRPV pathway in OA, encompassing its involvement in pain perception, inflammation, and mechanotransduction. Furthermore, we discuss the latest research findings, potential therapeutic strategies, and future directions in the field, shedding light on the multifaceted nature of TRPV channels in OA.

## 1. Introduction to Osteoarthritis

Osteoarthritis (OA), one of the most common types of arthritis, is a chronic degenerative and disabling disease characterized by complex disease of the entire synovial joint [1], including structural defects in the hyaliphatic articular cartilage, loss of intact subchondral bone, meniscal degeneration, infrapatellar fat pad and synovial membrane inflammation and fibrosis, and instability of tendons and ligaments [2,3,4]. OA can involve multiple joints, including the hip, knee, ankle, hand, temporomandibular joint (TMJ), and other joints [1,5,6], among which the knee, hand, and hip are the most prone to OA [7]. The estimated global incidence of symptomatic osteoarthritis is 240 million people, including 10% of men and 18% of women aged 60 years and older [2]. In 2021, 22% of adults over the age of 40 had knee OA, and it is estimated that more than 500 million people are currently affected by OA globally [8]. According to a large cohort study in the United States, the prevalence of knee osteoarthritis (KOA) has increased 2.1-fold since the 1950s [9]. The prevalence of OA is expected to increase from 26.6% to 29.5% by 2032 [10].

OA is the main cause of disability worldwide, and is a significant burden on the medical and social economy [11,12,13]. Its clinical manifestations include joint pain, movement disorder, stiffness, and appearance distortion, which lead to great pain and inconvenience to patients [1,2,14]. The pathogenesis of OA is still unclear, but many studies have stated that the pathological changes in OA involve the whole joint [1,2,3,4]. At the same time, the pathological changes in joint tissue lead to the production of proinflammatory factors, which further stimulate and injure the receptors, resulting in hyperalgesia. Current medical technology has not been able to cure osteoarthritis, although various treatment options [15], such as medications, physical therapy, and lifestyle modifications, aim to manage symptoms, improve joint function, and enhance the quality of life for affected individuals. However, OA with severe symptoms can only eventually be treated with joint replacement. Joint replacement surgery is increasing at a rate of 10% per year worldwide, and 95% of these surgeries are performed on patients with OA [16]. Though joint replacement is an important method for the treatment of OA, which can effectively relieve joint pain, restore joint function, and improve the quality of life of patients, patients must bear the high cost and high risk of joint replacement surgery [17]. Therefore, there is an urgent need for a new strategy for the treatment of OA that can delay the progression of OA via early prevention before the large-scale destruction of articular cartilage occurs.

## 2. Etiology of Osteoarthritis

The etiology of osteoarthritis involves various factors, including aging, joint injuries, female gender, obesity, genetics, and lifestyle [18,19,20].

Aging has been recognized as the most important risk factor for OA. Although aging and OA can be completely independent processes, they are closely related from a statistical point of view [21,22]. In recent years, several possible pathological mechanisms of aging leading to OA have been proposed, for example, joint tissues naturally undergoing degeneration and repair processes, leading to their decreased efficiency with age [23]. Greene et al. [24] showed that joint tissue cells, including chondrocytes and meniscus cells, as well as infrapatellar fat adjacent to the knee joint, may be a local source of inflammatory mediators that increase with age and lead to OA. At the same time, increased production of proinflammatory mediators such as cytokines and chemokines, as well as matrix-degrading enzymes important for joint tissue destruction, may be a consequence of cellular senescence and the development of the senescence-associated secretory phenotype (SASP). In addition, Blanco et al. [25] and Hui et al. [26] showed that oxidative stress induced by age-related mitochondrial dysfunction, characterized by excessive accumulation of reactive oxygen species (ROS) and imbalance of energy metabolism in articular chondrocytes, may promote articular chondrocyte apoptosis and articular cartilage destruction.

Joint damage is another major risk factor for OA. Felson et al. [27] suggested that most or almost all aspects of OA are caused by mechanical damage to the joint tissue. Clinical statistics in the United States predict that traumatic OA accounts for 12–42% of OA (the proportion varies by age), and the actual proportion may be higher [16]. Recently, Laitner et al. [28] showed that traumatized joints are five times more likely to develop OA than untraumatized joints. In recent years, the role of mechanical stress in the pathogenesis of OA has also been a research hotspot, including the pattern, force, and duration of joint stress [29,30]. However, the molecular mechanisms of how mechanical stress contributes to the occurrence and progression of OA need to be studied in more detail to guide the clinical physiotherapy of OA patients.

It is well known that women are more susceptible to OA than men [7,31]. It has been shown that 18% of the female population aged 60 years and older suffer from symptomatic OA compared to only 10% of the male population [2,8]. In addition, some recent studies [28,31,32] have shown that female OA patients have more severe joint pathological changes and clinical symptoms compared with male OA patients. Although these studies have suggested that this gender difference may be caused by the reduction in sex hormone levels in menopausal women, the underlying factors leading to this gender difference in the development of OA are still not fully understood [28,31,32].

Obesity is another major risk factor for hip and knee OA. The reason may be that overweight and obesity leads to a significant increase in mechanical load of hip and knee joints and articular cartilage wear and tear accompanied by ligament destruction in patients, and eventually leads to the occurrence of OA. Of note, obese patients also have a higher incidence of OA in the non-weight-bearing hand [33,34]. The reason may be that cytokines such as retinol binding protein 4 (RBP4), leptin, adiponectin, resistin, and other cytokines released by a large number of adipocytes in obese patients promote the occurrence and progression of OA [35,36,37]. In addition, the increased production of enzymes, oxidative stress, and the release of proinflammatory cytokines can lead to cartilage degradation and inflammation within the joint [38].

In addition, genetics plays a significant role in the development of OA [39]. In the past, studies have confirmed that genetic factors are associated with OA in humans. A study by Boer et al. [40] in 2021 confirmed that genetics is a risk factor for 11 types of OA including hand, hip, and spine. In this study, the authors performed a genome-wide association study meta-analysis of 826,690 people (177,517 with osteoarthritis) from 13 international cohorts from nine populations, and identified 100 independently associated risk variants across 11 osteoarthritis phenotypes. Although some genetic studies [39,41,42] have identified effector genes that affect the development of OA, these genetic data still need to be further validated and studied.

## 3. Signal Pathways and Ion Channels Related to Osteoarthritis

In the past few decades, the pathogenesis of OA has been extensively studied [43], but the complex ion channels and signaling pathways involved in the pathogenesis and development of OA are still not fully understood. Previous studies have confirmed that the signaling pathways involved in OA include the Wnt pathway [44,45,46,47,48], Notch pathway [49,50,51], AMPK pathway [52,53,54], nuclear factor-κb (NF-κB) pathway [55,56,57,58,59], MAPKs pathway [15,60,61,62], Hippo-YAP pathway [63], TGF-β/Smad pathway [4,64,65], mTOR pathway [66,67,68], and the OPG/RANK/RANKL pathway [69,70]. In addition, the ion channel theory has been increasingly investigated in the pathogenesis of arthritis. Common related mechanosensitive channels include the degenerin/epithelial sodium channel (DEG/ENaC), transient receptor potential channel (TRP), two-pore-domain potassium channel (K2P), and the Piezo channel [71]. Among them, the TRP and Piezo channels are mechanosensitive ion channels that have been widely studied. These ion channels can respond to various mechanical stimuli such as gravity, fluid shear stress, compressive stress, and tensile force [72].

In recent years, a growing number of studies have shown that TRP channels play anti-inflammatory and analgesic roles in joints while participating in the maintenance of their normal physiological functions [73,74,75,76,77,78]. For example, transient receptor potential vanilloid-1 (TRPV1) has been found to be an important transducer of chemical, inflammatory, and neuropathic pain signals, and is expressed in a variety of neuronal and non-neuronal tissues and organs, including chondrocytes, fibroblasts, macrophages, and the dorsal root ganglion (DRG), which play an important role in inflammatory diseases such as OA and rheumatoid arthritis [73,74]. Transient receptor potential vanilloid-4 (TRPV4) has been shown to respond directly or indirectly to a variety of mechanical signals, such as stretch, compression, osmotic pressure, and shear stress [75]. Recently, functional changes in TRPV1 and TRPV4 channels have been identified as risk factors for OA, and their abnormal expression and function can cause cell necrosis and apoptosis, cartilage extracellular matrix degradation, synovial inflammatory response, and hyperalgesia [76,77,78], suggesting that TRPV1 and TRPV4 play an important role in OA pain generation as well as disease progression. Therefore, TRPV is expected to become a new therapeutic target for the treatment of OA. It is of great significance to study the expression and mechanism of TRPV in OA, and to develop TRPV-related drugs to inhibit OA pain and structural destruction. This article comprehensively summarizes the expression and mechanism of TRPV channels in OA as well as the progress of TRPV-channel-related drugs in OA treatment, in order to provide new insights and ideas for the clinical treatment of OA in the future.

## 4. The TRPV Channel Family Overview

TRPV channels are part of the TRP channel superfamily, and are named for their sensitivity to vanillic acid and capsaicin [79,80,81]. TRPV channels are multimodal proteins gated by a variety of mechanisms that are structurally similar to other TRP channels, but contain an additional 3–5 ankyrin repeat domain at the N-terminus [82]. This N-terminal anchoring protein repeat domain is one of the important clues for understanding the functional diversity of TRPV channels [83]. The TRPV subfamily consists of TRPV1–6 members, which are divided into two subgroups: TRPV1–4 and TRPV5–6 [84]. TRPV1–4 can be activated by heat, and are therefore referred to as thermal TRP channels [85]. They form homogeneous and heterogeneous channels that show mild Ca^2+^ selectivity [86]. However, TRPV5 and TRPV6 can form homologous and heteromeric channels that are highly selective for Ca^2+^ [87,88]. Although the TRPV family members are highly similar in sequence, each member exhibits specific expression and biological functions.

TRPV1 channels are expressed in both neuronal and non-neuronal cells, and there is evidence that TRPV1 channels play a key role in various physiological functions [89]. TRPV1 is highly expressed in small-diameter sensory neurons such as the DRG, trigeminal ganglia, and vagal ganglia [90,91], and is mainly localized in the plasma membrane [92,93]. TRPV1 channels can be blocked by noxious heat (>42 °C) [94], animal peptide toxins, fatty acids, active compounds (such as capsaicin [90]), and other irritants [95] activated and involved in thermal nociception [96,97,98]. Caterina et al. [97] showed that knockout TRPV1-/-mice exhibited reduced postinflammatory thermal hyperalgesia and impaired nociception. In addition, Plant et al. [96] showed similar results where heat-induced pain perception was impaired in TRPV1 KO mice. Thus, TRPV1 agonists induce pain-related behaviors in mice, whereas the pain-related behaviors are attenuated in TRPV1 null mice. Drug blockades have shown analgesic effects in various pain models, including arthritis and cancer pain [99]. TRPV1 is also involved in the physiological function of non-neuronal tissues, and studies have found that non-neuronal expression is seen in the arteriolar smooth muscle of the skin and trachea [100,101]. In addition, TRPV1 plays a role in respiratory tract functions such as coughing [102]. In smooth muscle and endothelial cells, it contributes to the control of chronic hypoxia-induced vasculature [103], and in keratinocytes it may be involved in skin proliferation promoted by low pH [104].

TRPV2 is widely expressed in almost all cell types, but is particularly highly expressed in the DRG, brain, lung, and spleen [92,105]. Under normal physiological conditions, TRPV2 channels are mainly located in the inner membrane of the cell [106,107]. However, after stimulation by growth factors (IGF-I), hormones, cytokines, and endocannabinoids, they translocate from the inner membrane to the plasma membrane [105,108]. TRPV2 has a variety of physiological roles, ranging from sensing noxious stimuli to nociception. TRPV2 exerts its function mainly by acting as a lipid sensor, thermal sensor, and mechanical sensor to regulate intracellular calcium homeostasis [109,110]. In addition, TRPV2 function is regulated by phosphatidylinositol 4, 5-bisphosphate (PIP2) and extracellular signal-regulated kinase (ERK)-dependent phosphorylation [107,111]. TRPV2 acts as a nonspecific cation channel activated by noxious temperatures of 52 °C to regulate thermal nociception, and responds to low osmolality, stretch, and shear stress in tissues other than the brain [112]. Aguettaz et al. [113] found that TRPV2 was highly expressed in cardiomyocytes and involved in the stretch-dependent response of the heart. Similarly, Naticchioni et al. [114] showed that mice lacking TRPV2 exhibited impaired cardiac function. TRPV2 plays an important role in maintaining Ca^2+^ homeostasis in vascular smooth muscle and cardiomyocytes [115]. Overstimulation of TRPV2 has been implicated in muscular dystrophy, myocardial fibrosis, and cardiomyopathy caused by Ca^2+^ overload [116,117]. In addition, the expression and normal function of TRPV2 in different types of immune cells were confirmed, and macrophages showed an especially high expression rate of TRPV2 [110,118]. Link et al. [119] observed impaired macrophage activation in TRPV2-deficient mice, resulting in increased susceptibility to bacterial infection. In addition, Santoni et al. [120] reported that TRPV2 was overexpressed in different types of cancer.

TRPV3 is widely expressed in a variety of tissues, including the brain, sensory neurons, DRG, spinal cord, tongue, nose, palate, colon, and testis [93,121], and especially in epithelial cells and keratinocytes of skin [122,123]. TRPV3 is a Ca^2+^-permeable, nonselective, temperature-sensitive cation channel that responds to noxious heat, with a threshold of approximately 39 °C in skin keratinocytes [124]. Activation of TRPV3 can regulate a variety of downstream functions, including skin barrier formation, wound healing, temperature perception, pruritus, and pain [125,126]. Thus, TRPV3 is essential for normal skin barrier formation [127]. On the other hand, excessive activation of TRPV3 can lead to skin damage and increase the risk of dermatitis [128]. Recently, Zhong et al. [129] showed that a gain in function mutation in the TRPV3 gene can cause Olmsted syndrome, which is characterized by progressive hyperkeratosis leading to hand and foot damage, inflammatory dermatitis infiltration, abnormal hair growth, pruritus, and pain. Notably, Moqrich et al. [130] found that TRPV3 KO mice did not respond to harmless/noxious heat. Cheng et al. [127] further found that TRPV3^−/−^ gene knockout mice showed wavy ear flaps, curly whiskers, and changes in temperature preference.

In contrast to other thermal TRPV channels, TRPV4 has been implicated in a number of channel diseases, suggesting a broad expression pattern and multiple physiological functions. TRPV4 is highly expressed in the musculoskeletal tissues, kidney, heart, liver, spleen, brain, sensory neurons, skin, epithelial cells, urinary tract, and vascular system [131,132,133]. TRPV4 is a Ca^2+^-permeable, nonselective cation channel that is expressed in a variety of tissues, and responds to stimuli such as temperature, mechanical stress, hypotonic conditions, and small-molecule compounds [134]. TRPV4 directs various physiological functions in different cell types through mediated Ca^2+^ influx, and plays an important role in cell volume regulation [135,136]. For example, it is expressed in vascular endothelial cells and contributes to the regulation of vascular tone and blood pressure [137]. In mammary epithelial tissue, TRPV4 specifically regulates the integrity of cell–cell junctions through the expression of tight junction proteins [138]. TRPV4 regulates osmotic balance by regulating water secretion in the kidney, and contributes to renal function [139]. In the musculoskeletal system, TRPV4 is required for mechanical stress transduction [140]. In addition, TRPV4 protects the skin barrier [141], regulation of hair follicle growth [142], and pruritus in some pathologies [143]. Interestingly, TRPV4^−/−^ mice showed only slight changes in temperature response, while other physiological functions were significantly more affected [144]. Knockout mice have impaired bladder function, increased bladder volume, increased bone mass, impaired vascular endothelial function, and impaired pressure and pain perception [145,146]. In contrast to the mild phenotypic alterations in mice, more than 50 TRPV4 gene mutations were detected. These mutations cause a variety of diseases in human patients, such as different forms of skeletal dysplasia, neuropathy, and muscular atrophy [147].

TRPV5 and TRPV6 share 75% amino acid sequence identity; thus, they are both highly selective for Ca^2+^, sensitive to 1,25-dihydroxy-vitamin D3 (1,25-(OH)_2_-VitD3), and play important roles in maintaining Ca^2+^ homeostasis and regulating bone metabolism [87,88]. TRPV5 was mainly expressed in the distal convoluted tubule (DCT) and connecting tubule (CNT) of the kidney [148,149], and its mRNA was found in human placenta, osteoclasts, and lymphocytes in low amounts [150,151]. However, TRPV6 is significantly expressed in a variety of tissues, including the gastrointestinal tract, kidney, placenta, breast, pancreas, testis, and prostate, especially in prostate cancer and most other common cancers [109]. Several recent studies [152,153,154,155,156] have also confirmed that TRPV5 and TRPV6 play important roles in maintaining Ca^2+^ homeostasis and regulating bone metabolism. Hoenderop et al. [152] showed that TRPV5 gene knockout mice exhibited dysfunction of Ca^2+^ reabsorption and severe hypercalciuria, followed by significant abnormalities in bone structure. Subsequently, Renkema et al. [153] further confirmed that TRPV5^−/−^mice exhibited strong urinary Ca^2+^ excretion, followed by a significant increase in serum concentrations of calcitriol and parathyroid hormone (PHT) as a compensatory mechanism. As a result of the elevated concentrations of these hormones, a decrease in bone mineral density and increased Ca^2+^ reabsorption in the GI tract were observed [154]. TRPV6 contributes to intestinal Ca^2+^ absorption [155], and Bianco et al. [156] found that TRPV6 gene knockout mice had reduced bone mineral density and increased serum Ca^2+^ concentration. The basis for maintaining normal bone metabolic activity is to maintain a dynamic balance between bone formation by osteoblasts and bone resorption by osteoclasts. Osteoblasts are the most important functional cells in the process of bone formation and development, and also regulate osteoclasts to a certain extent [157,158]. Ca^2+^ transport in and out of cells is closely related to these bone metabolic processes [158]. Therefore, a certain degree of dynamic balance must be maintained between blood calcium and bone calcium.

In summary, TRPVs serve as sensors for different stimuli (heat, mechanical stress, and cytokines); play diverse and critical roles in both physiological and pathological processes in most tissues; and contribute to the homeostasis of electrolytes, the maintenance of barrier functions, and the development of macrophages (Table 1). Consequently, investigating the functions and regulatory mechanisms of these channels is significant for understanding the pathogenesis of related diseases and developing novel therapeutic strategies.

## 5. The Expression and Role of TRPV Channels in Osteoarthritis

In summary, TRPV ion channels are widely distributed in various systems and tissues of the human body. TRPV ion channels are a class of cation channels that can respond to a variety of stimuli such as harmful heat, hormones, cytokines, small-molecule compounds, and mechanical stress, and are involved in a variety of signaling pathways. Therefore, in the context of osteoarthritis, it is of great significance to further study their expression and mechanism of action in OA, and to develop TRPV-related drugs to inhibit OA pain, inflammation, and structural destruction.

### 5.1. TRPV1

TRPV1, as a widely distributed ion channel in the nervous system, is highly expressed in nerve terminals such as the DRG, trigeminal ganglia, and vagal ganglia [90,91,171]. There is evidence of a key role in various physiological functions [89] and a crucial role in the context of osteoarthritis [76,77,78]. In the investigation conducted by Cho et al. [172], advanced tracing and immunocytochemistry techniques were employed to demonstrate the presence of TRPV1-positive fibers innervating both knee and ankle joints in mice. Their study revealed widespread expression of TRPV1 channels in the synovium, cartilage, and nerve fibers associated with joints, emphasizing the crucial role of TRPV1 in detecting and transmitting pain signals related to joint conditions.

Throughout the pathological progression of osteoarthritis, the release of inflammatory factors activates TRPV1 channels, thereby triggering an increase in neuronal excitability and intensifying the transmission of pain signals [173]. The activation of TRPV1 channels could lead to the release of inflammatory mediators, such as hormones and cytokines, contributing to the development and persistence of joint pain and inflammation [174]. Kochukov et al. [175] proposed that TRP channels are functionally expressed in human synoviocytes, potentially playing a crucial role in adaptive or pathological alterations of joint surfaces during arthritis inflammation. Subsequent studies in articular cartilage showed that exposure of chondrocytes to the inflammatory factors IL-1β and TNF-α resulted in increased TRPV1 expression in chondrocytes [176]. Another study found that TNF-α could regulate TRPV4 through the p38MAPK inflammatory pathway [177]. Spahn et al. [178] delved into the sensitization of TRPV1 via activation of TRPA1, involving adenylyl cyclase, increased cAMP, subsequent translocation and activation of PKA, and phosphorylation of TRPV1 at PKA phosphorylation residues.

In addition, TRPV1 is expressed in some immune cells, and is a novel regulator of the immune system [179,180]. Lv et al. [181] observed a simultaneous increase in TRPV1 expression and M1-type macrophage infiltration in human and rat OA synovia. Their results indicate that TRPV1 inhibits the polarization of M1 macrophages in the synovium through the Ca^2+^/CaMKII/Nrf2 signaling pathway, thereby reducing the progression of OA. In addition, Engler et al. [182] demonstrated the expression of TRPV1 in synovial fibroblasts (SFs) from patients with symptomatic OA and rheumatoid arthritis (RA). Stimulation of cultured OA-SF and RA-SF with the TRPV1 agonist capsaicin resulted in increased expressions of IL-6 mRNA and IL-6 protein in the cell culture supernatant. This suggests a non-neuronal role for TRPV1 in regulating nociception in symptomatic OA and RA patients. A recent study by DEWAKER et al. [183] in 2023 further elaborated the important role of TRPV1 in the detection of noxious stimuli (heat, acid, capsaicin). Its role in pain makes it a potential drug target for the treatment of chronic pain, migraines, and related disorders.

**Figure 1:**
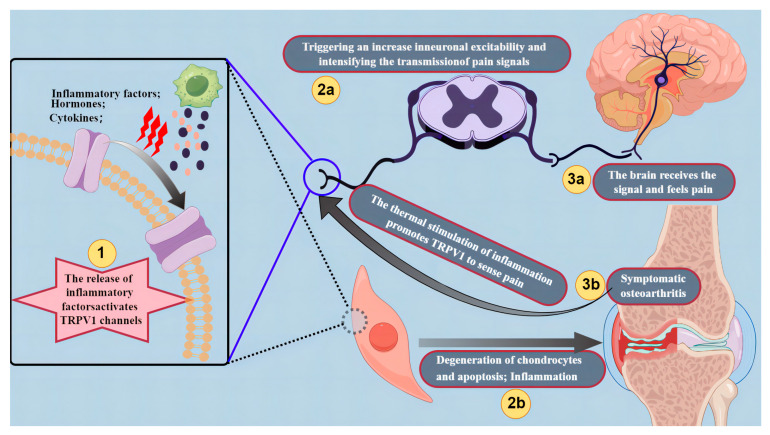
The roles of TRPV1 channels in osteoarthritis. (1, 2a, 3a) The release of inflammatory factors activates TRPV1 channels, thereby triggering an increase in neuronal excitability and intensifying the transmission of pain signals. (1, 2b, 3b) The activation of TRPV1 channels could lead to the release of inflammatory mediators, such as hormones and cytokines, contributing to the development and persistence of joint pain and inflammation. Furthermore, thermal stimulation during inflammation enhances TRPV1 sensitivity in the pain pathway. Created by Figdraw.com (https://www.figdraw.com).

Overall, the involvement of TRPV1 in inflammatory responses, particularly its responsiveness to thermal stimuli, establishes it as a pivotal nexus linking pain and inflammation [184]. In osteoarthritis, TRPV1 primarily expressed in sensory neurons contributes to pain perception and transmission. It becomes activated in response to inflammatory mediators, contributing to heightened pain sensitivity. Additionally, TRPV1 activation leads to the release of proinflammatory cytokines, further exacerbating the inflammatory response [185]. Therefore, the mechanisms of TRPV1 in osteoarthritis encompass its distribution within the nervous system and its intricate regulatory role in pain transmission and inflammatory responses (Figure 1).

These studies highlight the relevance of TRPV1 channels in arthritis, where they significantly contribute to the pain and inflammation characteristics of the disease, and contribute to our understanding of the intricate involvement of TRPV1 channels in the pathophysiology of joint disorders. These findings highlight the potential of targeting TRPV1, offering promising approaches for the development of therapeutic strategies for OA.

### 5.2. TRPV2

As a nonspecific cation channel, TRPV2 is widely expressed in almost all cell types, and has a variety of physiological roles ranging from sensing noxious stimuli to pain sensation [92,109]. Therefore, exploring the expression and role of TRPV2 in osteoarthritis will elucidate the molecular mechanism of osteoarthritis.

In a seminal study conducted by Nakamoto et al. [186], an exploration into the expression of TRPV2 in both mouse and human articular cartilage, as well as ectopic ossification lesions, revealed noteworthy insights into the regulatory mechanisms governing articular cartilage. The study underscored the pivotal role of TRPV2 in this context, demonstrating its influence through the induction of Prg4 and the simultaneous suppression of ectopic ossification. This compelling evidence strongly suggests that TRPV2 plays a crucial protective role in the maintenance of joint health. Furthermore, it proposes the potential of TRPV2 as a viable target for innovative approaches to osteoarthritis treatment. In a parallel vein, Laragione et al. [187] delved into the intricate web of signaling molecules within fibroblast-like synoviocytes (FLSs) derived from rheumatoid arthritis patients. Their investigation brought to light a novel facet of TRPV2 function, namely its ability to suppress the activation of specific signaling molecules such as Rac1 and RhoA. This finding implies a significant regulatory role of TRPV2 in key processes related to cell invasion in the context of arthritis. Consequently, the study posits TRPV2 as a potential therapeutic target for addressing the intricate mechanisms underlying this debilitating condition.

By synthesizing these findings, it becomes evident that TRPV2 emerges as a multifaceted player in joint health, exerting protective effects in the realm of articular cartilage maintenance and demonstrating regulatory potential in the context of arthritis. The studies by Nakamoto et al. [186] and Laragione et al. [187] collectively contribute to a growing body of knowledge that positions TRPV2 as a promising avenue for further exploration in the quest for innovative osteoarthritis treatments.

### 5.3. TRPV3

TRPV3 is a Ca^2+^-permeable, non-selective, and temperature-sensitive cation channel [124]. When activated, TRPV3 can regulate a variety of downstream functions, including skin barrier formation, wound healing, temperature perception, pruritus, and pain [125,126]. However, its expression and function in osteoarthritis are still unclear.

In the investigation conducted by Somogyi et al. [188], the mRNA expressions of TRPV1, TRPV2, TRPV3, TRPV4, and TRPV6 were identified in high-density cartilage cultures established from the limb buds of chicken and mouse embryos. Notably, the expression pattern of TRPVs underwent a switch during chondrogenesis in both cultures. Inhibition of TRPVs with nonselective calcium channel blockers not only reduced chondrogenesis, but also significantly inhibited proliferation. Furthermore, incubating cell cultures at 41 °C resulted in increased TRPV1 expression and enhanced cartilage matrix production. Additionally, elevated mRNA levels of TRPV3 were detected during the differentiation of chondrocytes into matrix-producing chondrocytes. These findings collectively demonstrate that TRPV1 and TRPV3 expressions are responsive to thermal and mechanical stimuli, respectively. As a result, these channels emerge as potential candidates contributing to the transduction of environmental stimuli in chondrogenic cells. This study provides valuable insights into the dynamic regulation of TRPV expression during chondrogenesis, and highlights the roles of TRPV1 and TRPV3 in responding to specific environmental cues, thereby influencing the behavior of chondrogenic cells. The research by Somogyi et al. [188] contributes to our understanding of the intricate interplay between TRPV channels and environmental stimuli in the context of cartilage development.

### 5.4. TRPV4

TRPV4 is a Ca^2+^-permeable, nonselective cation channel that is expressed in a variety of tissues, and responds to stimuli such as temperature, mechanical stress, hypotonic conditions, and small-molecule compounds [134]. Previous studies have shown that the functional change in the TRPV4 channel is considered a risk factor for OA, and it plays an important role in the production of OA pain and disease progression [76,77,78].

As an intracellular second messenger, Ca^2+^ has been implicated in the survival and functional expression of chondrocytes. Previous studies have shown that inflammatory factors such as interleukin-1β (IL-1β) and tumor necrosis factor-α (TNF-α) can promote TRPA1 mRNA and protein expression and channel opening; this causes a large influx of Ca^2+^ into the cell, but eventually leads to chondrocyte apoptosis due to intracellular Ca^2+^ overload and mitochondrial dysfunction [189,190]. Activation of TRPV4 channels promotes the expressions of cartilage marker SRY-related high mobility group-box 9 (Sox9), aggrecan (ACAN), and collagen II (COL-II) through the Ca^2+^/calmodulin (CaM) signaling pathway [191]. SOX9 plays an important role in chondrogenesis, differentiation, and functional maintenance of chondrocytes. Thus, TRPV4 serves as a key regulator of chondrogenesis. Recent studies have confirmed that TRPV4 channels affect the formation and development of bone and joint diseases by regulating Ca^2+^ homeostasis. For example, the study of Muramatsu et al. [192] showed that activation of TRPV4 increased the steady-state expression of SOX9 mRNA and protein, as well as SOX6 mRNA, suggesting its role in regulating the SOX9 pathway and promoting the chondrogenesis process. Clark et al. [193] delved into the changes in osteoarthritis and bone structure by examining knee joints in TRPV4^−/−^ mice at different age intervals. The loss of TRPV4 resulted in a deficiency in osmotically induced calcium signaling in articular chondrocytes, accompanied by a progressive, sex-dependent increase in bone mineral density and osteoarthritis joint degeneration. The presence of TRPV4 in articular chondrocytes and its involvement in the response to low osmolality are mediated by the release of extracellular Ca^2+^ and intracellular Ca^2+^. Moreover, TRPV4 was implicated in regulating the production or influence of proinflammatory molecules on the osmotic response [194]. Masuyama et al. [195], in experiments with TRPV4 (R616Q/V620I) transgenic mice, revealed that activation of TRPV4 in osteoclasts regulates Ca^2+^/calmodulin signaling, increasing the number of osteoclasts and their resorptive activity, and leading to bone loss. Itoh et al. [167] conducted experimental studies on synoviocytes from patients with rheumatoid arthritis (RA) and non-rheumatoid arthritis (CTR), revealing TRPV4 as a novel regulator of intracellular Ca^2+^ in human synoviocytes. In addition, the mutations in TRPV4 cation channels can cause various osteoarticular diseases, including skeletal dysplasia with severe dwarfism and bone mineral density and structural abnormalities [168].

Biomechanical factors play a key role in the pathogenesis of OA, especially mechanical stress. Chondrocytes transform mechanical stimulation signals into cellular metabolic and biosynthetic activities through mechanoreceptor-mediated mechanotransduction, thereby affecting cartilage growth and development and the degenerative process of OA [196,197]. Mechanical loading in the form of cyclic stretch could activate TRPV4 channels in articular chondrocytes, thereby inhibiting IL-1β-induced nitric oxide (NO) and prostaglandin E2 (PGE2) release [198]. The production of inflammatory mediators, such as NO and PGE2, can lead to cartilage degeneration in OA. Thus, TRPV4 plays an important regulatory role in chondrocyte inflammatory signal transduction. Khatib et al. [199] demonstrated that TRPV4 plays a crucial role in prenatal osteojoint development, influencing cartilage growth and joint formation by responding to mechanical stimuli. TRPV4 protein expression in both osteoblasts and osteoclasts, as well as TRPV4 deficiency suppressed reduced levels of mineral deposition and bone formation due to unloading, indicating TRPV4 plays a key role in unloading-induced bone loss [200]. O’Conor et al. [201] verified the high expression of TRPV4 in articular chondrocytes, and emphasized its central role in transducing mechanical signals, supporting the maintenance of the cartilage extracellular matrix, and joint health. In 2022, Zhang et al. [202] further explained that TRPV4 was involved in the sensing of mechanical and inflammatory signals in chondrocytes, and introduced the important role of TRPV4 in regulating the mechanical conduction of various functions of chondrocytes in the biomechanical microenvironment. In summary, advances in understanding the complex role of TRPV4-mediated mechanical signaling mechanisms hold promise for reproducing the physio-biomechanical microenvironment and designing biomaterials with cell-inducing effects for cartilage tissue engineering, and targeting TRPV4-mediated mechanotransduction was proposed as a potential strategy for treating diseases such as osteoarthritis.

Furthermore, studies have shown that M1 macrophages are considered to be the main cause of pathological changes in OA tissues, which include osteophyte formation and synovitis [203]. Activation of TRPV4 channels in synoviocytes leads to the generation of reactive oxygen species (ROS), which oxidize protein and lipid components and cause synoviocyte apoptosis in OA, and excessive ROS production promotes the production of interleukins and MMPs, thereby accelerating the degradation of the extracellular matrix [204]. Sun et al. [204] showed that inhibition of TRPV4 in a rat OA model could effectively alleviate cartilage damage, synovitis, and osteophyte formation, and also reduces the number of M1 macrophages in the synovium. In vitro studies also found that blocking TRPV4 channels in RAW264.7 cells could inhibit the ROS/NLRP3 signaling pathway, thereby reducing the polarization of M1 macrophages to inhibit the progression of OA.

**Figure 2:**
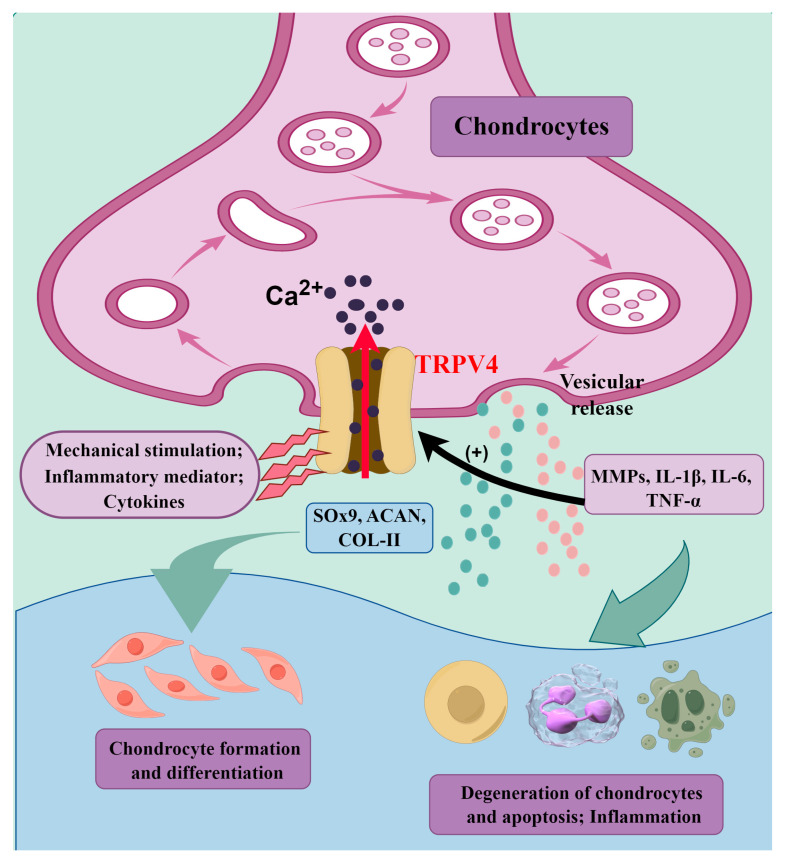
Mechanisms of TRPV4 channels in osteoarthritis. Inflammatory factors such as IL-1β and TNF-α can promote TRPV4 channel opening; this causes a large influx of Ca^2+^ into the cell. Activation of TRPV4 channels promotes the expressions of Sox9, ACAN, and COL-II through the Ca^2+^/calmodulin (CaM) signaling pathway; this promotes chondrocyte formation and differentiation. However, the activation of TRPV4 channels leads to the release of inflammatory mediators (MMPs, IL-1β, IL-6, and TNF-α), contributing to the development and persistence of joint pain and inflammation. Created by Figdraw.com (https://www.figdraw.com).

These studies collectively contribute to our comprehensive understanding of the multifaceted role of TRPV4 in joint health, cartilage development, and bone homeostasis. The findings also suggest potential therapeutic implications for targeting TRPV4 in conditions such as osteoarthritis, and for enhancing matrix formation in tissue-engineered cartilage (Figure 2).

### 5.5. TRPV5

TRPV5 is highly selective for Ca^2+^, and plays an important role in maintaining Ca^2+^ homeostasis [87,88]. TRPV5 is mainly expressed in the kidney, where it regulates Ca^2+^ reabsorption [148,149]. Previous studies [152] have shown that TRPV5 knockout mice exhibit reduced bone mineral density and significant abnormalities in bone structure. Zhou et al. [205] conducted a comprehensive examination of TRPV5 expression in articular chondrocytes under normal and exercise loading conditions. Their results revealed ubiquitous expression of TRPV5 in all osteochondral tissues, with its expression level dependent on bone and joint loading. This suggests a potential role for TRPV5 in the formation and development of cartilage tissue. In a related study, Chen et al. [206] investigated the protective effects of magnesium sulfate on cartilage in rabbits, implicating autophagy initiation mechanisms potentially linked to TRPV5. This suggests that reducing TRPV5 activity could be beneficial for cartilage health and regeneration, offering insights into therapeutic approaches for conditions like post-traumatic osteoarthritis (PTOA). Examining the influence of culture and passage times on TRPV4, TRPV5, and TRPV6 expressions in articular chondrocytes, Hdud et al. [207] found that these channels were consistently expressed across all passages, with TRPV5 and TRPV6 showing upregulation over time and passages. These findings suggest the potential involvement of these TRPV channels in calcium signaling and homeostasis in chondrocytes. More recently, Wei et al. [208,209] observed upregulation of TRPV5 expression in chondrocytes of osteoarthritic rats, implicating TRPV5 as a crucial initiator of exogenous chondrocyte apoptosis. The mechanism involves upregulated TRPV5 activating CaMKII phosphorylation by regulating Ca^2+^ influx, subsequently impacting chondrocyte apoptosis through MAPK and Akt/mTOR pathways.

These studies collectively shed light on the intricate role of TRPV5 in cartilage biology and pathology, suggesting its potential as a therapeutic target for conditions like osteoarthritis. The findings contribute to our understanding of the molecular mechanisms underlying cartilage homeostasis and disease progression, opening avenues for further research into innovative treatment strategies.

### 5.6. TRPV6

Like TRPV5, TRPV6 is highly selective for Ca^2+^ and plays an important role in maintaining Ca^2+^ homeostasis by regulating intestinal Ca^2+^ absorption [87,88,155]. Furthermore, TRPV6 knockout mice were found to have reduced bone mineral density [156]. Song et al. [210] conducted an analysis of the expression level of TRPV6 in both an osteoarthritis (OA) rat model and knee cartilage obtained from OA patients. Additionally, they explored bone structure and observed osteoarthritis changes in the knee joints of TRPV6 gene knockout mice. The findings revealed a significant downregulation of TRPV6 expression in the OA rat model. Furthermore, TRPV6 knockout mice exhibited pronounced osteoarthritis changes, characterized by cartilage fibrillation, eburnation, and loss of proteoglycans. Notably, the depletion of TRPV6 had a substantial impact on chondrocyte functions, influencing extracellular matrix secretion, release of matrix-degrading enzymes, as well as cell proliferation and apoptosis.

These results collectively underscore the potential role of TRPV6 in the pathogenesis of osteoarthritis, shedding light on its involvement in maintaining cartilage integrity and regulating key cellular processes within chondrocytes. The research by Song et al. [210] contributes valuable insights into the molecular mechanisms underlying osteoarthritis, presenting TRPV6 as a potential target for further exploration in the development of therapeutic interventions for this debilitating condition.

In summary, with the deepening of research, significant progress has been made in the research of the TRPV pathway in osteoarthritis. For example, researchers have developed animal models for osteoarthritis to simulate the disease’s progression [211]. These models are used to validate the precise role of the TRPV pathway in pain perception, inflammation, and joint destruction. These studies provide crucial information on how the TRPV pathway affects the entire disease process. Together, these results highlight the potential role of TRPV channels in the pathogenesis of osteoarthritis, revealing their involvement in maintaining cartilage integrity and regulating key cellular processes within chondrocytes. Therefore, TRPV is expected to become a new therapeutic target for the treatment of OA, and the continued search for TRPV-related drugs to inhibit OA pain, inflammation, and structural destruction is of great significance for the treatment of OA (Table 2).

## 6. Potential Treatment Strategies of TRPV Channels in Osteoarthritis

Taken together, TRPV channels play important roles in the formation and progression of OA. Recent advancements in pharmacology have shown promise in selectively targeting TRPV channels to manage pain, inflammation, and other conditions [212]. Therefore, the use of TRPV as a drug target for OA has therapeutic potential. It is noteworthy that TRPV1 and TRPV4 have attracted more attention from scholars in the field due to their special distributions and roles in OA.

The identification of TRPV channels as potential therapeutic targets for OA has spurred the development of specific modulators and drugs [213]. Recently, Logashina et al. [214] investigated the anti-inflammatory properties of APHC3, a peptide modulator of TRPV1, in a model of OA induced by sodium iodoacetate (MIA), and in a model of RA induced by complete Fredrin adjuvant (CFA). Comparisons were also made with commonly used non-steroidal anti-inflammatory drugs (NSAIDs) such as diclofenac, ibuprofen, and meloxicam. The results showed that the analgesic and anti-inflammatory effects of APHC3 were equal to or better than those of NSAIDs. APHC3 also significantly reversed joint swelling, disability, grip strength impairment, and thermal and mechanical hypersensitivity reactions. Long-term treatment can reduce the concentration of IL-1b in synovial fluid, alleviate the inflammatory changes in joints, and prevent cartilage degeneration. Therefore, peptide APHC3 has the potential to be an analgesic and anti-inflammatory substance for relieving arthritis symptoms. In addition, the dual-acting compound OMDM198 (FAAH inhibitor/TRPV1 antagonist) reversed the effects of MIA-induced OA on spinal cords in rats, restoring Alox12, Mapk14, and Prkcg to normal levels [215]. Meanwhile, a study by Atobe et al. [216] in 2019 reported that intra-articular injection of TRPV4 quinazolin-4 (3H)-one derivative 36·HCl (36·HCl) enhanced the expressions of ACAN and SOX9 mRNA in the articular cartilage of OA rats to inhibit cartilage degradation, indicating that 36·HCl promoted chondrocyte anabolism, and thus inhibited the progression of OA. In addition, studies have found that the TRPV4 activator 4α-phorbol 12,13-didecanoate (4α-PDD) acts on arthritic synovial cells to inhibit the production of inflammatory cytokines, helping to prevent the occurrence or further deterioration of OA [167]. Oxoglaucine protects against cartilage damage by blocking the TRPV5/CAMK-II/calmodulin pathway to inhibit Ca^2+^ influx and activate autophagy [169,170]. The TRPV4 inhibitor GSK2193874 inhibited the upregulation of calmodulin and caspase-8 and the apoptosis of chondrocytes in the rat OA anterior cruciate ligament transection model [217]. Intra-articular administration of the selective TRPV4 antagonist HC067047 reduces cartilage extracellular matrix loss, cartilage wear, and osteophyte formation [204]. The discovery of TRPV channel modulators lays the foundation for the targeted therapy of TRPV in OA.

In recent years, several TRPV modulators from chemical, biological, and natural sources have entered clinical trials, but most of the previous TRPV antagonist projects were shelved due to the high incidence of adverse reactions such as hyperthermia and paresthesia. In 2017, Brown et al. [218] found that the most commonly reported adverse reactions after treatment with NEO6860 were fever, headache, numbness, nausea, and dizziness in a clinical phase I double-blind, placebo-controlled, dose-escalation study involving 64 subjects. In this study, both a single oral dose of 800 mg and two oral doses of 500 mg (12 h apart) of NEO6860 were well tolerated. Unlike other TRPV1 antagonists, despite comprehensive and specific monitoring of body temperature and thermal pain threshold/tolerance, no significant clinical elevations in these parameters were found. Pharmacodynamic parameters (induced pain and secondary hypoalgesia) were improved at 3 and 8 h after administration of NEO6860. These findings indicate that NEO6860 is a TRPV1 antagonist that only blocks capsaicin activation. In addition, in 2019, Stevens et al. [219] evaluated the efficacy and safety of high-purity synthetic trans-capsaicin (CNTX-4975) in a phase II multicenter double-blind study in patients with chronic moderate to severe osteoarthritis (OA)-related knee pain. In this study, a total of 172 patients with KOA were randomized in a 2:1:2 ratio to receive a single intra-articular injection of placebo, CNTX-4975 0.5 mg, or CNTX-4975 1.0 mg. The results showed a greater decrease in pain score AUC in the CNTX-4975 0.5 mg and 1.0 mg groups than in the placebo group at week 12, and the significant improvement in the 1.0 mg group was maintained at week 24. No safety concerns were identified during the study, which indicates that the incidence of adverse events was similar in all treatment groups. The results of this study support the efficacy and safety of intra-articular injection of trans-capsaicin for the treatment of moderate to severe pain associated with KOA. Subsequently, Stevens [220] conducted an 8-week, open-label, phase III clinical study (NCT03661996) on the efficacy of CNTX-4975 in OA in 2020, in which 848 KOA patients with pain symptoms underwent intra-knee injections with 1mg of CNTX-4975. The results showed that the magnitude of pain reduction was similar to the results of a double-blind randomized controlled trial reported previously. These targeted modulators of TRPV1 can relieve the symptoms of OA while avoiding the disadvantages of previous TRPV antagonists that produce obvious side effects, providing new ideas and directions for the targeted therapy of TRPV channels in OA.

The clinical development of TRPV channel modulators provides a scientific basis and new insights for further utilization of TRVP channels as biomarkers for OA treatment. Despite the attractiveness of TRPV channels in the clinical treatment of pain and inflammation, several challenges remain. In clinical studies, some common adverse reactions of TRPV channels in the treatment of OA, such as high fever, heat burn, cold, hypertension, headache, nausea, abnormal taste, and paresthesia, are difficulties for researchers in this field. For example, common adverse effects in subjects who received oral NEO6860, a TRPV1 antagonist, included fever, headache, paresthesia, nausea, and dizziness [218]. Patients treated with ABT-102, a TRPV1 channel antagonist, experienced adverse effects such as blunted sensation and altered taste [221,222]. In addition, patients treated with the TRPV1 channel antagonist JNJ-39439335 were found to have reduced thermal sensation, paresthesia, and mild burns to the skin that felt cold [223,224].

In conclusion, TRPV channels are involved in the physiological and pathophysiological processes of OA, making them promising targets for pharmacological intervention. However, the molecular gating mechanism of TRPV channels in response to different stimuli in the joint environment is still unclear, and the widely distributed characteristics of TRPV channels often cause cross-interactions in vivo and lead to adverse reactions, which limits the application of TRPV-channel-targeted drugs in OA treatment. Therefore, further studies on highly selective TRPV agonists and antagonists for in vivo applications are needed to reduce cross-interactions caused by TRPV channels and reduce damage to other tissues (Table 3).

## 7. Future Research Directions for TRPV Channels in Osteoarthritis

Future osteoarthritis research will continue to focus on the role of the TRPV pathway. Areas of focus may include more in-depth mechanistic studies, larger clinical trials, and the search for new TRPV pathway inhibitors. These studies will help improve the treatment and management of osteoarthritis.

Future research should aim to develop more precise modulators and regulators that target specific TRPV channel subtypes. Understanding the unique roles of each subtype and their effects on different conditions; investigating the detailed mechanisms of TRPV channel activation, desensitization, and regulation; and understanding how these channels function at a molecular level are essential for developing targeted therapies. Structural biology studies can be continued to gain insights into the three-dimensional structures of TRPV channels. This knowledge will aid in designing more effective drugs and modulators.

In addition, these areas hold promise for novel therapeutic interventions for TRPV channels in the treatment of osteoarthritis. For example, expanding the clinical applications of TRPV modulation includes conducting further clinical trials to assess the safety and efficacy of TRPV-based therapies for various conditions such as pain management, neuroinflammatory disorders, and musculoskeletal diseases. The potential synergistic effects of combining TRPV-based therapies with existing treatments may also be investigated. Exploring combination therapies may enhance treatment outcomes for a range of conditions. Expanding research into the role of TRPV channels in neuroinflammatory conditions, neurodegenerative diseases, and neuropathic pain, as well as advancing personalized medicine approaches to tailor TRPV-based treatments to individual patients, are further potential research paths. This involves identifying patient-specific factors that influence treatment responses. Exploring the impact of diet and nutrition on TRPV channel activity and understanding how dietary components affect TRPV channels can lead to dietary interventions for managing related conditions. Investigating how environmental factors, such as temperature and humidity, influence TRPV-mediated conditions may enable environmental modifications for symptom management. Gene therapies that target TRPV channels can be developed, potentially offering long-term and highly targeted treatment options. Conducting comprehensive studies on the safety and potential side effects of TRPV channel modulators is also a possibility. Understanding their long-term effects is crucial for clinical implementation. Cross-disciplinary collaboration between researchers should be encouraged in fields like molecular biology, pharmacology, physiology, and clinical medicine to foster a holistic understanding of TRPV channels.

In summary, understanding the role of TRPV channels in osteoarthritis and their potential as therapeutic targets is a promising avenue for future research. Such studies may lead to innovative treatments and a better grasp of the mechanisms underlying this prevalent joint disorder.

## Figures and Tables

**Table 1:** The functions of TRPV channels.

Channels	Distribution	Functions	Pathological Roles	Related Diseases	References
TRPV1	Brain, DRG, Sensory nerve, Liver, Kidney, Brain, Urinary bladder, Pancreas, Testis	Mechanosensitivity; Thermosensitivity; Neurodepolarization	Inflammatory pain; Neuropathic pain; Aberrant thermosensitivity	Osteoarthritis; Migraine; ARDS; Fibromyalgia	[96,97,98,99,159,160,161,162]
TRPV2	Brain, DRG, Sensory nerve, Spinal cord, Liver, Lung, Spleen, Muscle, Intestine, Urinary bladder, Immune cells	Thermosensitivity; Thermosensitivity; Cell cycle regulation	Cell proliferation abnormalities; Tumor growth; Mechanical injury sensitivity	Heart failure	[75,91,105,109,110,120,163]
TRPV3	Keratinocytes, Brain, DRG, Sensory nerve, Spinal cord, Skin, Tongue, Nose, Palate, Colon, Testicles	Thermosensitivity; Maintain normal skin barrier	Skin injuries; Pain symptoms; Sensory abnormalities	Dermatitis; Psoriatic lesions; Olmsted syndrome	[75,93,123,127,128,129,164,165]
TRPV4	Musculoskeletal tissue, Brain, Skin, Sensory neurons, DRG, Kidney, Liver, Lung, Spleen, Heart, Vascular endothelia	Mechanosensitivity; Thermosensitivity; Regulates the musculoskeletal system	Inflammation; Pain perception; Cellular deformation; Cell proliferation abnormalities	Osteoarthritis; Skeletal dysplasia; Neuromuscular disorders	[131,132,133,134,140,147,166,167,168]
TRPV5	Kidney, Placenta, Pancreas, Prostate	Maintenance of Ca^2+^ homeostasis; Renal regulation	Calcium metabolism abnormalities; Renal diseases	Nephrolithiasis; Osteoporosis;	[75,88,148,149,152,153,154,169,170]
TRPV6	Intestine, Kidney, Pancreas, Breast, Placenta, Testes, Prostate	Maintenance of Ca^2+^ homeostasis; Intestinal calcium regulation	Calcium metabolism abnormalities; Intestinal inflammation	Osteoporosis; Hyperparathyroidism; Cancer	[75,88,109,155,156,169,170]

**Table 2:** The expressions of TRPV channels in osteoarthritis.

Channels	Distribution	Species	Results/Conclusions	References
TRPV1	Synovium; Cartilage; Nerve fibers associated with joints	Mouse	TRPV1 positive fibers regulate knee and ankle pain sensation in mice	[172]
	Synovium	Synoviocytes; Patients with inflammatory arthropathies	TRP1 channels are functionally expressed in human synoviocytes	[175]
	Synovium	Rats and patients with OA	TRPV1 expression and M1 macrophage infiltration were simultaneously increased in both human and rat OA synovia	[181]
	Synovial fibroblasts	Patients with symptomatic OA and RA	TRPV1 is expressed in SF from symptomatic OA and RA patients	[182]
TRPV2	Articular cartilage Ectopic ossification lesions	Mouse; Human	Regulation of articular cartilage by TRPV2 through Prg4 induction and suppression of ectopic ossification	[186]
	Fibroblast-Like Synoviocyte (FLS)	Patients with RA	Stimulation of TRPV2 in FLS is capable of suppressing the activation of RhoA and Rac1	[187]
TRPV3	Cartilage	Chicken and mouse embryos	The mRNA level of TRPV3 was increased	[188]
TRPV4	Cartilage	Murine induced pluripotent stem cells (iPSCs)	TRPV4 serves both as a marker and a regulator of iPSC chondrogenesis	[191]
	Cartilage	TRPV4^(−/−)^ mice	Deletion of TRPV4 leads to osteoarthritic joint degeneration	[193]
	Cartilage	Murine chondrogenic cell line (ATDC5)	TRPV4 regulates the SOX9 pathway and contributes to the process of chondrogenesis	[192]
	Cartilage	Porcines	TRPV4 is expressed in articular chondrocytes and mediates the hypoosmotic response	[194]
	Cartilage	Embryonic mouse	TRPV4 promotes joint cartilage growth and morphogenesis	[199]
	Synovium	Patients with RA	TRPV4 is a functional regulator of Ca^2+^ in human synoviocytes	[167]
	Synovium	Tumor-derived SW982 synoviocytes; Patients with inflammatory arthropathies	TRPV4 may play a critical role in adaptive or pathological changes in articular surfaces during arthritic inflammation	[175]
	Bone	Trpv4(R616Q/V620I) transgenic mice	Activation of TRPV4 can promote sufficient of osteoclast function	[195]
	Bone	Wild-type mice	TRPV4 protein is expressed in both osteoblasts and osteoclasts	[200]
TRPV5	Cartilage	Normal and OA SD rats	TRPV5 is expressed in all cartilage tissues	[205]
	Cartilage	Rabbit	Reducing TRPV5 activity could be beneficial for cartilage health and regeneration	[206]
	Cartilage	Rats with osteoarthritis	Upregulated TRPV5 expression was observed in chondrocytes from rats with osteoarthritis	[209]
TRPV6	Cartilage	OA rat and OA patients; TRPV6 knockout mice	TRPV6 as a cartilage protective factor was involved in the pathogenesis of OA	[210]

**Table 3:** Clinical trials of TRPV channel-targeted therapy for osteoarthritis.

Channels	Drug	Clinical Progress	Function	Adverse Events	Organization/NCT Number	References
TRPV1	CNTX-4975	Phase II/III	Reduce pain	/	NCT02558439; NCT03661996	[219,220,225]
	Resiniferatoxin (RTX)	Phase I/II/III	Reduce pain and improve mobility	Destroys nerve endings produces reversible analgesia	NCT02566564 2018-000818-37(EU); NCT04044742	[225,226,227]
	AZD1386	Phase II	/	No significant pain decrease	NCT00878501	[225,227,228]
	JNJ-39439335	Phase I/II	Analgesic	Thermal hypoesthesia; Paresthesia; feeling cold; minor thermal burns	NCT01006340 NCT00933582 NCT01343303	[223,224,225,227]
	ABT-102	Phase I	Analgesic	Dysesthesias; altered taste sensation	NCT00854659	[221,225,227]
	NEO6860	Phase I	Analgesic	Feeling of hotness; headache; paresthesia; nausea; dizziness	NCT02337543	[218,225,227]

## References

[1] Hunter D.J., Bierma-Zeinstra S. (2019). Osteoarthritis. Lancet.

[2] Allen K.D., Thoma L.M., Golightly Y.M. (2022). Epidemiology of osteoarthritis. Osteoarthr. Cartil..

[3] Kang D., Shin J., Cho Y., Kim H.S., Gu Y.R., Kim H., You K.T., Chang M.J., Chang C.B., Kang S.B. (2019). Stress-activated miR-204 governs senescent phenotypes of chondrocytes to promote osteoarthritis development. Sci. Transl. Med..

[4] Yao Q., Wu X., Tao C., Gong W., Chen M., Qu M., Zhong Y., He T., Chen S., Xiao G. (2023). Osteoarthritis: Pathogenic signaling pathways and therapeutic targets. Signal Transduct. Target. Ther..

[5] Lu K., Ma F., Yi D., Yu H., Tong L., Chen D. (2022). Molecular signaling in temporomandibular joint osteoarthritis. J. Orthop. Transl..

[6] Leung Y.Y., Thumboo J. (2011). An overview of OA research in two urban APLAR populations. Int. J. Rheum. Dis..

[7] Prieto-Alhambra D., Judge A., Javaid M.K., Cooper C., Diez-Perez A., Arden N.K. (2014). Incidence and risk factors for clinically diagnosed knee, hip and hand osteoarthritis: Influences of age, gender and osteoarthritis affecting other joints. Ann. Rheum. Dis..

[8] Quicke J.G., Conaghan P.G., Corp N., Peat G. (2022). Osteoarthritis year in review 2021: Epidemiology & therapy. Osteoarthr. Cartil..

[9] Wallace I.J., Worthington S., Felson D.T., Jurmain R.D., Wren K.T., Maijanen H., Woods R.J., Lieberman D.E. (2017). Knee osteoarthritis has doubled in prevalence since the mid-20th century. Proc. Natl. Acad. Sci. USA.

[10] Turkiewicz A., Petersson I.F., Björk J., Hawker G., Dahlberg L.E., Lohmander L.S., Englund M. (2014). Current and future impact of osteoarthritis on health care: A population-based study with projections to year 2032. Osteoarthr. Cartil..

[11] Liu Q., Niu J., Li H., Ke Y., Li R., Zhang Y., Lin J. (2017). Knee Symptomatic Osteoarthritis, Walking Disability, NSAIDs Use and All-cause Mortality: Population-based Wuchuan Osteoarthritis Study. Sci. Rep..

[12] Lo J., Chan L., Flynn S. (2021). A Systematic Review of the Incidence, Prevalence, Costs, and Activity and Work Limitations of Amputation, Osteoarthritis, Rheumatoid Arthritis, Back Pain, Multiple Sclerosis, Spinal Cord Injury, Stroke, and Traumatic Brain Injury in the United States: A 2019 Update. Arch. Phys. Med. Rehabil..

[13] Long H., Zeng X., Liu Q., Wang H., Vos T., Hou Y., Lin C., Qiu Y., Wang K., Xing D. (2020). Burden of osteoarthritis in China, 1990-2017: Findings from the Global Burden of Disease Study 2017. Lancet Rheumatol..

[14] Silverwood V., Blagojevic-Bucknall M., Jinks C., Jordan J.L., Protheroe J., Jordan K.P. (2015). Current evidence on risk factors for knee osteoarthritis in older adults: A systematic review and meta-analysis. Osteoarthr. Cartil..

[15] Gu Y.-T., Chen J., Meng Z.-L., Ge W.-Y., Bian Y.-Y., Cheng S.-W., Xing C.-K., Yao J.-L., Fu J., Peng L. (2017). Research progress on osteoarthritis treatment mechanisms. Biomed. Pharmacother..

[16] Little C.B., Hunter D.J. (2013). Post-traumatic osteoarthritis: From mouse models to clinical trials. Nat. Rev. Rheumatol..

[17] Charlesworth J., Fitzpatrick J., Perera N.K.P., Orchard J. (2019). Osteoarthritis—A systematic review of long-term safety implications for osteoarthritis of the knee. BMC Musculoskelet. Disord..

[18] Warner S.C., Valdes A.M. (2016). The Genetics of Osteoarthritis: A Review. J. Funct. Morphol. Kinesiol..

[19] Roos E.M., Lohmander L.S. (2003). The Knee injury and Osteoarthritis Outcome Score (KOOS): From joint injury to osteoarthritis. Health Qual. Life Outcomes.

[20] Lieberthal J., Sambamurthy N., Scanzello C.R. (2015). Inflammation in joint injury and post-traumatic osteoarthritis. Osteoarthr. Cartil..

[21] O’brien M.S., Mcdougall J.J. (2019). Age and frailty as risk factors for the development of osteoarthritis. Mech. Ageing Dev..

[22] Loeser R.F., Collins J.A., Diekman B.O. (2016). Ageing and the pathogenesis of osteoarthritis. Nat. Rev. Rheumatol..

[23] Deshpande B.R., Katz J.N., Solomon D.H., Yelin E.H., Hunter D.J., Messier S.P., Suter L.G., Losina E. (2016). Number of Persons with Symptomatic Knee Osteoarthritis in the US: Impact of Race and Ethnicity, Age, Sex, and Obesity. Arthritis Care Res..

[24] Greene M.A., Loeser R.F. (2015). Aging-related inflammation in osteoarthritis. Osteoarthr. Cartil..

[25] Blanco F.J., Rego I., Ruiz-Romero C. (2011). The role of mitochondria in osteoarthritis. Nat. Rev. Rheumatol..

[26] Hui W., Young D.A., Rowan A.D., Xu X., Cawston T.E., Proctor C.J. (2016). Oxidative changes and signalling pathways are pivotal in initiating age-related changes in articular cartilage. Ann. Rheum. Dis..

[27] Felson D.T. (2013). Osteoarthritis as a disease of mechanics. Osteoarthr. Cartil..

[28] Laitner M.H., Erickson L.C., Ortman E. (2021). Understanding the Impact of Sex and Gender in Osteoarthritis: Assessing Research Gaps and Unmet Needs. J. Women’s Health.

[29] Astephen Wilson J.L., Kobsar D. (2021). Osteoarthritis year in review 2020: Mechanics. Osteoarthr. Cartil..

[30] Andersson J.K., Hagert E., Brittberg M. (2021). Cartilage Injuries and Posttraumatic Osteoarthritis in the Wrist: A Review. Cartilage.

[31] Peshkova M., Lychagin A., Lipina M., Di Matteo B., Anzillotti G., Ronzoni F., Kosheleva N., Shpichka A., Royuk V., Fomin V. (2022). Gender-Related Aspects in Osteoarthritis Development and Progression: A Review. Int. J. Mol. Sci..

[32] Tschon M., Contartese D., Pagani S., Borsari V., Fini M. (2021). Gender and Sex Are Key Determinants in Osteoarthritis Not Only Confounding Variables. A Systematic Review of Clinical Data. J. Clin. Med..

[33] Raud B., Gay C., Guiguet-Auclair C., Bonnin A., Gerbaud L., Pereira B., Duclos M., Boirie Y., Coudeyre E. (2020). Level of obesity is directly associated with the clinical and functional consequences of knee osteoarthritis. Sci. Rep..

[34] Reyes C., Leyland K.M., Peat G., Cooper C., Arden N.K., Prieto-Alhambra D. (2016). Association Between Overweight and Obesity and Risk of Clinically Diagnosed Knee, Hip, and Hand Osteoarthritis: A Population-Based Cohort Study. Arthritis Rheumatol..

[35] Jiang H., Pu Y., Li Z.H., Liu W., Deng Y., Liang R., Zhang X.M., Zuo H.D. (2022). Adiponectin, May Be a Potential Protective Factor for Obesity-Related Osteoarthritis. Diabetes Metab. Syndr. Obes. Targets Ther..

[36] Dai Y., Zhang L., Yan Z., Li Z., Fu M., Xue C., Wang J. (2021). A low proportion n-6/n-3 PUFA diet supplemented with Antarctic krill (Euphausia superba) oil protects against osteoarthritis by attenuating inflammation in ovariectomized mice. Food Funct..

[37] Sekar S., Wu X., Friis T., Crawford R., Prasadam I., Xiao Y. (2018). Saturated fatty acids promote chondrocyte matrix remodeling through reprogramming of autophagy pathways. Nutrition.

[38] Antony B., Singh A. (2021). Imaging Biochem. Markers Osteoarthr..

[39] Aubourg G., Rice S.J., Bruce-Wootton P., Loughlin J. (2022). Genetics of osteoarthritis. Osteoarthr. Cartil..

[40] Boer C.G., Hatzikotoulas K., Southam L., Stefánsdóttir L., Zhang Y., Coutinho de Almeida R., Wu T.T., Zheng J., Hartley A., Teder-Laving M. (2021). Deciphering osteoarthritis genetics across 826,690 individuals from 9 populations. Cell.

[41] Young D.A., Barter M.J., Soul J. (2022). Osteoarthritis year in review: Genetics, genomics, epigenetics. Osteoarthr. Cartil..

[42] Ratneswaran A., Kapoor M. (2021). Osteoarthritis year in review: Genetics, genomics, epigenetics. Osteoarthr. Cartil..

[43] Tong L., Yu H., Huang X., Shen J., Xiao G., Chen L., Wang H., Xing L., Chen D. (2022). Current understanding of osteoarthritis pathogenesis and relevant new approaches. Bone Res..

[44] Nalesso G., Thomas B.L., Sherwood J.C., Yu J., Addimanda O., Eldridge S.E., Thorup A.S., Dale L., Schett G., Zwerina J. (2017). WNT16 antagonises excessive canonical WNT activation and protects cartilage in osteoarthritis. Ann. Rheum. Dis..

[45] Tong W., Zeng Y., Chow D.H.K., Yeung W., Xu J., Deng Y., Chen S., Zhao H., Zhang X., Ho K.K. (2019). Wnt16 attenuates osteoarthritis progression through a PCP/JNK-mTORC1-PTHrP cascade. Ann. Rheum. Dis..

[46] Zhu M., Chen M., Zuscik M., Wu Q., Wang Y.J., Rosier R.N., O’Keefe R.J., Chen D. (2008). Inhibition of beta-catenin signaling in articular chondrocytes results in articular cartilage destruction. Arthritis Rheum..

[47] Li Y., Xiao W., Sun M., Deng Z., Zeng C., Li H., Yang T., Li L., Luo W., Lei G. (2016). The Expression of Osteopontin and Wnt5a in Articular Cartilage of Patients with Knee Osteoarthritis and Its Correlation with Disease Severity. BioMed Res. Int..

[48] Zhou Y., Wang T., Hamilton J.L., Chen D. (2017). Wnt/β-catenin Signaling in Osteoarthritis and in Other Forms of Arthritis. Curr. Rheumatol. Rep..

[49] Liu Z., Chen J., Mirando A.J., Wang C., Zuscik M.J., O’Keefe R.J., Hilton M.J. (2015). A dual role for NOTCH signaling in joint cartilage maintenance and osteoarthritis. Sci. Signal..

[50] Monteagudo S., Lories R.J. (2018). A Notch in the joint that exacerbates osteoarthritis. Nat. Rev. Rheumatol..

[51] Zanotti S., Yu J., Bridgewater D., Wolf J.M., Canalis E. (2018). Mice harboring a Hajdu Cheney Syndrome mutation are sensitized to osteoarthritis. Bone.

[52] Ge Y., Zhou S., Li Y., Wang Z., Chen S., Xia T., Shen J., Teng H., Jiang Q. (2019). Estrogen prevents articular cartilage destruction in a mouse model of AMPK deficiency via ERK-mTOR pathway. Ann. Transl. Med..

[53] Mével E., Shutter J.A., Ding X., Mattingly B.T., Williams J.N., Li Y., Huls A., Kambrath A.V., Trippel S.B., Wagner D. (2022). Systemic inhibition or global deletion of CaMKK2 protects against post-traumatic osteoarthritis. Osteoarthr. Cartil..

[54] Qiu L., Tang C.L., Huang S.Q., Zhao D.D., Luo A., Wu M.J., An H.Y., Tan C.F., Yang Z.X., Zhu Z.W. (2018). Effect of Electroacupuncture on Synovial M 1/M 2 Macrophage Polarization in Rats with Acute Gouty Arthritis. Acupunct. Res..

[55] Caron M.M., Emans P.J., Surtel D.A., Cremers A., Voncken J.W., Welting T.J., van Rhijn L.W. (2012). Activation of NF-κB/p65 facilitates early chondrogenic differentiation during endochondral ossification. PLoS ONE.

[56] Nakatomi C., Nakatomi M., Matsubara T., Komori T., Doi-Inoue T., Ishimaru N., Weih F., Iwamoto T., Matsuda M., Kokabu S. (2019). Constitutive activation of the alternative NF-κB pathway disturbs endochondral ossification. Bone.

[57] Ulivi V., Giannoni P., Gentili C., Cancedda R., Descalzi F. (2008). p38/NF-kB-dependent expression of COX-2 during differentiation and inflammatory response of chondrocytes. J. Cell. Biochem..

[58] Chen S., Qin L., Wu X., Fu X., Lin S., Chen D., Xiao G., Shao Z., Cao H. (2020). Moderate Fluid Shear Stress Regulates Heme Oxygenase-1 Expression to Promote Autophagy and ECM Homeostasis in the Nucleus Pulposus Cells. Front. Cell Dev. Biol..

[59] Rigoglou S., Papavassiliou A.G. (2013). The NF-κB signalling pathway in osteoarthritis. Int. J. Biochem. Cell Biol..

[60] Li Z., Dai A., Yang M., Chen S., Deng Z., Li L. (2022). p38MAPK Signaling Pathway in Osteoarthritis: Pathological and Therapeutic Aspects. J. Inflamm. Res..

[61] Zhang Y., Pizzute T., Pei M. (2014). A review of crosstalk between MAPK and Wnt signals and its impact on cartilage regeneration. Cell Tissue Res..

[62] Gao S.-C., Yin H.-B., Liu H.-X., Sui Y.-H. (2014). Research progress on MAPK signal pathway in the pathogenesis of osteoarthritis. Zhongguo Gu Shang.

[63] Deng Y., Lu J., Li W., Wu A., Zhang X., Tong W., Ho K.K., Qin L., Song H., Mak K.K. (2018). Reciprocal inhibition of YAP/TAZ and NF-κB regulates osteoarthritic cartilage degradation. Nat. Commun..

[64] Zhong L., Huang X., Karperien M., Post J.N. (2015). The Regulatory Role of Signaling Crosstalk in Hypertrophy of MSCs and Human Articular Chondrocytes. Int. J. Mol. Sci..

[65] Gamer L.W., Pregizer S., Gamer J., Feigenson M., Ionescu A., Li Q., Han L., Rosen V. (2018). The Role of Bmp2 in the Maturation and Maintenance of the Murine Knee Joint. J. Bone Miner. Res. Off. J. Am. Soc. Bone Miner. Res..

[66] Pal B., Endisha H., Zhang Y., Kapoor M. (2015). mTOR: A potential therapeutic target in osteoarthritis?. Drugs RD.

[67] Zhang H., Wang H., Zeng C., Yan B., Ouyang J., Liu X., Sun Q., Zhao C., Fang H., Pan J. (2017). mTORC1 activation downregulates FGFR3 and PTH/PTHrP receptor in articular chondrocytes to initiate osteoarthritis. Osteoarthr. Cartil..

[68] Wu J., Kuang L., Chen C., Yang J., Zeng W.N., Li T., Chen H., Huang S., Fu Z., Li J. (2019). miR-100-5p-abundant exosomes derived from infrapatellar fat pad MSCs protect articular cartilage and ameliorate gait abnormalities via inhibition of mTOR in osteoarthritis. Biomaterials.

[69] Kovács B., Vajda E., Nagy E.E. (2019). Regulatory Effects and Interactions of the Wnt and OPG-RANKL-RANK Signaling at the Bone-Cartilage Interface in Osteoarthritis. Int. J. Mol. Sci..

[70] Muratovic D., Atkins G.J., Findlay D.M. (2023). Is RANKL a potential molecular target in osteoarthritis?. Osteoarthr. Cartil..

[71] Pei F., Liu J., Zhang L., Pan X., Huang W., Cen X., Huang S., Jin Y., Zhao Z. (2021). The functions of mechanosensitive ion channels in tooth and bone tissues. Cell. Signal..

[72] Cox C.D., Bavi N., Martinac B. (2019). Biophysical Principles of Ion-Channel-Mediated Mechanosensory Transduction. Cell Rep..

[73] Benítez-Angeles M., Morales-Lázaro S.L., Juárez-González E., Rosenbaum T. (2020). TRPV1: Structure, Endogenous Agonists, and Mechanisms. Int. J. Mol. Sci..

[74] Kim J.H. (2018). The Emerging Role of TRPV1 in Airway Inflammation. Allergy Asthma Immunol. Res..

[75] Sasase T., Fatchiyah F., Ohta T. (2022). Transient receptor potential vanilloid (TRPV) channels: Basal properties and physiological potential. Gen. Physiol. Biophys..

[76] Nummenmaa E., Hämäläinen M., Moilanen L.J., Paukkeri E.L., Nieminen R.M., Moilanen T., Vuolteenaho K., Moilanen E. (2016). Transient receptor potential ankyrin 1 (TRPA1) is functionally expressed in primary human osteoarthritic chondrocytes. Arthritis Res. Ther..

[77] Hinata M., Imai S., Sanaki T., Tsuchida J., Yoshioka T., Higashino K., Yamamoto M., Imai M., Soga M., Horita N. (2018). Sensitization of transient receptor potential vanilloid 4 and increasing its endogenous ligand 5,6-epoxyeicosatrienoic acid in rats with monoiodoacetate-induced osteoarthritis. Pain.

[78] Xing R., Wang P., Zhao L., Xu B., Zhang N., Li X. (2017). Mechanism of TRPA1 and TRPV4 Participating in Mechanical Hyperalgesia of Rat Experimental Knee Osteoarthritis. Arch. Rheumatol..

[79] Seebohm G., Schreiber J.A. (2021). Beyond Hot and Spicy: TRPV Channels and their Pharmacological Modulation. Cell. Physiol. Biochem. Int. J. Exp. Cell. Physiol. Biochem. Pharmacol..

[80] Tomohiro D., Mizuta K., Fujita T., Nishikubo Y., Kumamoto E. (2013). Inhibition by capsaicin and its related vanilloids of compound action potentials in frog sciatic nerves. Life Sci..

[81] Malfait A.-M., Miller R.J. (2016). Emerging Targets for the Management of Osteoarthritis Pain. Curr. Osteoporos. Rep..

[82] Venkatachalam K., Montell C. (2007). TRP channels. Annu. Rev. Biochem..

[83] Phelps C.B., Huang R.J., Lishko P.V., Wang R.R., Gaudet R. (2008). Structural analyses of the ankyrin repeat domain of TRPV6 and related TRPV ion channels. Biochemistry.

[84] Huynh K.W., Cohen M.R., Chakrapani S., Holdaway H.A., Stewart P.L., Moiseenkova-Bell V.Y. (2014). Structural insight into the assembly of TRPV channels. Structure.

[85] Satheesh N.J., Uehara Y., Fedotova J., Pohanka M., Büsselberg D., Kruzliak P. (2016). TRPV currents and their role in the nociception and neuroplasticity. Neuropeptides.

[86] Hellwig N., Albrecht N., Harteneck C., Schultz G., Schaefer M. (2005). Homo- and heteromeric assembly of TRPV channel subunits. J. Cell Sci..

[87] Zhang P., Sun C., Li H., Tang C., Kan H., Yang Z., Mao A., Ma X. (2018). TRPV4 (Transient Receptor Potential Vanilloid 4) Mediates Endothelium-Dependent Contractions in the Aortas of Hypertensive Mice. Hypertension.

[88] Na T., Peng J.B. (2014). TRPV5: A Ca(2+) channel for the fine-tuning of Ca(2+) reabsorption. Handb. Exp. Pharmacol..

[89] Szallasi A., Blumberg P.M. (1999). Vanilloid (Capsaicin) receptors and mechanisms. Pharmacol. Rev..

[90] Caterina M.J., Schumacher M.A., Tominaga M., Rosen T.A., Levine J.D., Julius D. (1997). The capsaicin receptor: A heat-activated ion channel in the pain pathway. Nature.

[91] Bujak J.K., Kosmala D., Szopa I.M., Majchrzak K., Bednarczyk P. (2019). Inflammation, Cancer and Immunity-Implication of TRPV1 Channel. Front. Oncol..

[92] Perálvarez-Marín A., Doñate-Macian P., Gaudet R. (2013). What do we know about the transient receptor potential vanilloid 2 (TRPV2) ion channel?. FEBS J..

[93] Peier A.M., Reeve A.J., Andersson D.A., Moqrich A., Earley T.J., Hergarden A.C., Story G.M., Colley S., Hogenesch J.B., McIntyre P. (2002). A heat-sensitive TRP channel expressed in keratinocytes. Science.

[94] Patapoutian A., Peier A.M., Story G.M., Viswanath V. (2003). ThermoTRP channels and beyond: Mechanisms of temperature sensation. Nat. Rev. Neurosci..

[95] Morales-Lázaro S.L., Serrano-Flores B., Llorente I., Hernández-García E., González-Ramírez R., Banerjee S., Miller D., Gududuru V., Fells J., Norman D. (2014). Structural determinants of the transient receptor potential 1 (TRPV1) channel activation by phospholipid analogs. J. Biol. Chem..

[96] Plant T.D. (2014). TRPs in mechanosensing and volume regulation. Handb. Exp. Pharmacol..

[97] Caterina M.J., Leffler A., Malmberg A.B., Martin W.J., Trafton J., Petersen-Zeitz K.R., Koltzenburg M., Basbaum A.I., Julius D. (2000). Impaired nociception and pain sensation in mice lacking the capsaicin receptor. Science.

[98] Cao E., Cordero-Morales J.F., Liu B., Qin F., Julius D. (2013). TRPV1 channels are intrinsically heat sensitive and negatively regulated by phosphoinositide lipids. Neuron.

[99] Brito R., Sheth S., Mukherjea D., Rybak L.P., Ramkumar V. (2014). TRPV1: A Potential Drug Target for Treating Various Diseases. Cells.

[100] Cavanaugh D.J., Chesler A.T., Jackson A.C., Sigal Y.M., Yamanaka H., Grant R., O’Donnell D., Nicoll R.A., Shah N.M., Julius D. (2011). Trpv1 reporter mice reveal highly restricted brain distribution and functional expression in arteriolar smooth muscle cells. J. Neurosci. Off. J. Soc. Neurosci..

[101] Kark T., Bagi Z., Lizanecz E., Pásztor E.T., Erdei N., Czikora A., Papp Z., Edes I., Pórszász R., Tóth A. (2008). Tissue-specific regulation of microvascular diameter: Opposite functional roles of neuronal and smooth muscle located vanilloid receptor-1. Mol. Pharmacol..

[102] Ho C.Y., Gu Q., Lin Y.S., Lee L.Y. (2001). Sensitivity of vagal afferent endings to chemical irritants in the rat lung. Respir. Physiol..

[103] Ristoiu V., Shibasaki K., Uchida K., Zhou Y., Ton B.T., Flonta M.L., Tominaga M. (2011). Hypoxia-induced sensitization of transient receptor potential vanilloid 1 involves activation of hypoxia-inducible factor-1 alpha and PKC. Pain.

[104] Denda M., Fuziwara S., Inoue K., Denda S., Akamatsu H., Tomitaka A., Matsunaga K. (2001). Immunoreactivity of VR1 on epidermal keratinocyte of human skin. Biochem. Biophys. Res. Commun..

[105] Kanzaki M., Zhang Y.Q., Mashima H., Li L., Shibata H., Kojima I. (1999). Translocation of a calcium-permeable cation channel induced by insulin-like growth factor-I. Nat. Cell Biol..

[106] Cohen M.R., Huynh K.W., Cawley D., Moiseenkova-Bell V.Y. (2013). Understanding the cellular function of TRPV2 channel through generation of specific monoclonal antibodies. PLoS ONE.

[107] Cohen M.R., Johnson W.M., Pilat J.M., Kiselar J., DeFrancesco-Lisowitz A., Zigmond R.E., Moiseenkova-Bell V.Y. (2015). Nerve Growth Factor Regulates Transient Receptor Potential Vanilloid 2 via Extracellular Signal-Regulated Kinase Signaling To Enhance Neurite Outgrowth in Developing Neurons. Mol. Cell. Biol..

[108] Liberati S., Morelli M.B., Amantini C., Santoni M., Nabissi M., Cardinali C., Santoni G. (2014). Advances in transient receptor potential vanilloid-2 channel expression and function in tumor growth and progression. Curr. Protein Pept. Sci..

[109] Kärki T., Tojkander S. (2021). TRPV Protein Family-From Mechanosensing to Cancer Invasion. Biomolecules.

[110] Shibasaki K. (2016). Physiological significance of TRPV2 as a mechanosensor, thermosensor and lipid sensor. J. Physiol. Sci. JPS.

[111] Mercado J., Gordon-Shaag A., Zagotta W.N., Gordon S.E. (2010). Ca2+-dependent desensitization of TRPV2 channels is mediated by hydrolysis of phosphatidylinositol 4,5-bisphosphate. J. Neurosci. Off. J. Soc. Neurosci..

[112] Sato M., Sobhan U., Tsumura M., Kuroda H., Soya M., Masamura A., Nishiyama A., Katakura A., Ichinohe T., Tazaki M. (2013). Hypotonic-induced stretching of plasma membrane activates transient receptor potential vanilloid channels and sodium-calcium exchangers in mouse odontoblasts. J. Endod..

[113] Aguettaz E., Bois P., Cognard C., Sebille S. (2017). Stretch-activated TRPV2 channels: Role in mediating cardiopathies. Prog. Biophys. Mol. Biol..

[114] Naticchioni M., Karani R., Smith M.A., Onusko E., Robbins N., Jiang M., Radzyukevich T., Fulford L., Gao X., Apel R. (2015). Transient Receptor Potential Vanilloid 2 Regulates Myocardial Response to Exercise. PLoS ONE.

[115] Koch S.E., Gao X., Haar L., Jiang M., Lasko V.M., Robbins N., Cai W., Brokamp C., Varma P., Tranter M. (2012). Probenecid: Novel use as a non-injurious positive inotrope acting via cardiac TRPV2 stimulation. J. Mol. Cell. Cardiol..

[116] Iwata Y., Ito S., Wakabayashi S., Kitakaze M. (2020). TRPV2 channel as a possible drug target for the treatment of heart failure. Lab. Investig. A J. Tech. Methods Pathol..

[117] Iwata Y., Katanosaka Y., Arai Y., Shigekawa M., Wakabayashi S. (2009). Dominant-negative inhibition of Ca2+ influx via TRPV2 ameliorates muscular dystrophy in animal models. Hum. Mol. Genet..

[118] Nagasawa M., Nakagawa Y., Tanaka S., Kojima I. (2007). Chemotactic peptide fMetLeuPhe induces translocation of the TRPV2 channel in macrophages. J. Cell. Physiol..

[119] Link T.M., Park U., Vonakis B.M., Raben D.M., Soloski M.J., Caterina M.J. (2010). TRPV2 has a pivotal role in macrophage particle binding and phagocytosis. Nat. Immunol..

[120] Santoni G., Amantini C., Maggi F., Marinelli O., Santoni M., Nabissi M., Morelli M.B. (2020). The TRPV2 cation channels: From urothelial cancer invasiveness to glioblastoma multiforme interactome signature. Lab. Investig. A J. Tech. Methods Pathol..

[121] Xu H., Ramsey I.S., Kotecha S.A., Moran M.M., Chong J.A., Lawson D., Ge P., Lilly J., Silos-Santiago I., Xie Y. (2002). TRPV3 is a calcium-permeable temperature-sensitive cation channel. Nature.

[122] Xu H., Delling M., Jun J.C., Clapham D.E. (2006). Oregano, thyme and clove-derived flavors and skin sensitizers activate specific TRP channels. Nat. Neurosci..

[123] Yan K., Sun X., Wang G., Liu Y., Wang K. (2019). Pharmacological Activation of Thermo-Transient Receptor Potential Vanilloid 3 Channels Inhibits Hair Growth by Inducing Cell Death of Hair Follicle Outer Root Sheath. J. Pharmacol. Exp. Ther..

[124] Smith G.D., Gunthorpe M.J., Kelsell R.E., Hayes P.D., Reilly P., Facer P., Wright J.E., Jerman J.C., Walhin J.P., Ooi L. (2002). TRPV3 is a temperature-sensitive vanilloid receptor-like protein. Nature.

[125] Aijima R., Wang B., Takao T., Mihara H., Kashio M., Ohsaki Y., Zhang J.Q., Mizuno A., Suzuki M., Yamashita Y. (2015). The thermosensitive TRPV3 channel contributes to rapid wound healing in oral epithelia. FASEB J. Off. Publ. Fed. Am. Soc. Exp. Biol..

[126] Miyamoto T., Petrus M.J., Dubin A.E., Patapoutian A. (2011). TRPV3 regulates nitric oxide synthase-independent nitric oxide synthesis in the skin. Nat. Commun..

[127] Cheng X., Jin J., Hu L., Shen D., Dong X.P., Samie M.A., Knoff J., Eisinger B., Liu M.L., Huang S.M. (2010). TRP channel regulates EGFR signaling in hair morphogenesis and skin barrier formation. Cell.

[128] Asakawa M., Yoshioka T., Matsutani T., Hikita I., Suzuki M., Oshima I., Tsukahara K., Arimura A., Horikawa T., Hirasawa T. (2006). Association of a mutation in TRPV3 with defective hair growth in rodents. J. Investig. Dermatol..

[129] Zhong W., Hu L., Cao X., Zhao J., Zhang X., Lee M., Wang H., Zhang J., Chen Q., Feng C. (2021). Genotype-Phenotype Correlation of TRPV3-Related Olmsted Syndrome. J. Investig. Dermatol..

[130] Moqrich A., Hwang S.W., Earley T.J., Petrus M.J., Murray A.N., Spencer K.S., Andahazy M., Story G.M., Patapoutian A. (2005). Impaired thermosensation in mice lacking TRPV3, a heat and camphor sensor in the skin. Science.

[131] Liu N., Wu J., Chen Y., Zhao J. (2020). Channels that Cooperate with TRPV4 in the Brain. J. Mol. Neurosci..

[132] Kassmann M., Harteneck C., Zhu Z., Nürnberg B., Tepel M., Gollasch M. (2013). Transient receptor potential vanilloid 1 (TRPV1), TRPV4, and the kidney. Acta Physiol..

[133] Filosa J.A., Yao X., Rath G. (2013). TRPV4 and the regulation of vascular tone. J. Cardiovasc. Pharmacol..

[134] Liedtke W., Choe Y., Martí-Renom M.A., Bell A.M., Denis C.S., Sali A., Hudspeth A.J., Friedman J.M., Heller S. (2000). Vanilloid receptor-related osmotically activated channel (VR-OAC), a candidate vertebrate osmoreceptor. Cell.

[135] Rosenbaum T., Benítez-Angeles M., Sánchez-Hernández R., Morales-Lázaro S.L., Hiriart M., Morales-Buenrostro L.E., Torres-Quiroz F. (2020). TRPV4: A Physio and Pathophysiologically Significant Ion Channel. Int. J. Mol. Sci..

[136] White J.P., Cibelli M., Urban L., Nilius B., McGeown J.G., Nagy I. (2016). TRPV4: Molecular Conductor of a Diverse Orchestra. Physiol. Rev..

[137] Liu L., Guo M., Lv X., Wang Z., Yang J., Li Y., Yu F., Wen X., Feng L., Zhou T. (2021). Role of Transient Receptor Potential Vanilloid 4 in Vascular Function. Front. Mol. Biosci..

[138] Islam M.A., Mizusawa M., Sharmin M.M., Hayashi S., Yonekura S. (2020). TRPV4 Increases the Expression of Tight Junction Protein-Encoding Genes via XBP1 in Mammary Epithelial Cells. Animals.

[139] Liedtke W., Friedman J.M. (2003). Abnormal osmotic regulation in trpv4-/- mice. Proc. Natl. Acad. Sci. USA.

[140] Corrigan M.A., Johnson G.P., Stavenschi E., Riffault M., Labour M.N., Hoey D.A. (2018). TRPV4-mediates oscillatory fluid shear mechanotransduction in mesenchymal stem cells in part via the primary cilium. Sci. Rep..

[141] Denda M., Sokabe T., Fukumi-Tominaga T., Tominaga M. (2007). Effects of skin surface temperature on epidermal permeability barrier homeostasis. J. Investig. Dermatol..

[142] Yang P., Lu P., Luo J., Du L., Feng J., Cai T., Yuan Y., Cheng H., Hu H. (2020). Transient stimulation of TRPV4-expressing keratinocytes promotes hair follicle regeneration in mice. Br. J. Pharmacol..

[143] Chen Y., Wang Z.L., Yeo M., Zhang Q.J., López-Romero A.E., Ding H.P., Zhang X., Zeng Q., Morales-Lázaro S.L., Moore C. (2021). Epithelia-Sensory Neuron Cross Talk Underlies Cholestatic Itch Induced by Lysophosphatidylcholine. Gastroenterology.

[144] Lee H., Iida T., Mizuno A., Suzuki M., Caterina M.J. (2005). Altered thermal selection behavior in mice lacking transient receptor potential vanilloid 4. J. Neurosci. Off. J. Soc. Neurosci..

[145] Sonkusare S.K., Bonev A.D., Ledoux J., Liedtke W., Kotlikoff M.I., Heppner T.J., Hill-Eubanks D.C., Nelson M.T. (2012). Elementary Ca^2+^ signals through endothelial TRPV4 channels regulate vascular function. Science.

[146] Masuyama R., Vriens J., Voets T., Karashima Y., Owsianik G., Vennekens R., Lieben L., Torrekens S., Moermans K., Vanden Bosch A. (2008). TRPV4-mediated calcium influx regulates terminal differentiation of osteoclasts. Cell Metab..

[147] Nilius B., Voets T. (2013). The puzzle of TRPV4 channelopathies. EMBO Rep..

[148] Peng J.B., Chen X.Z., Berger U.V., Vassilev P.M., Brown E.M., Hediger M.A. (2000). A rat kidney-specific calcium transporter in the distal nephron. J. Biol. Chem..

[149] Hoenderop J.G., Van Der Kemp A.W., Hartog A., van de Graaf S.F., van Os C.H., Willems P.H., Bindels R.J. (1999). Molecular identification of the apical Ca2+ channel in 1, 25-dihydroxyvitamin D3-responsive epithelia. J. Biol. Chem..

[150] Bernucci L., Henríquez M., Díaz P., Riquelme G. (2006). Diverse calcium channel types are present in the human placental syncytiotrophoblast basal membrane. Placenta.

[151] Van Der Eerden B.C., Hoenderop J.G., De Vries T.J., Schoenmaker T., Buurman C.J., Uitterlinden A.G., Pols H.A., Bindels R.J., van Leeuwen J.P. (2005). The epithelial Ca^2+^ channel TRPV5 is essential for proper osteoclastic bone resorption. Proc. Natl. Acad. Sci. USA.

[152] Hoenderop J.G., Van Leeuwen J.P., Van Der Eerden B.C., Kersten F.F., van der Kemp A.W., Mérillat A.M., Waarsing J.H., Rossier B.C., Vallon V., Hummler E. (2003). Renal Ca^2+^ wasting, hyperabsorption, and reduced bone thickness in mice lacking TRPV5. J. Clin. Investig..

[153] Renkema K.Y., Nijenhuis T., Van Der Eerden B.C., van der Kemp A.W., Weinans H., van Leeuwen J.P., Bindels R.J., Hoenderop J.G. (2005). Hypervitaminosis D mediates compensatory Ca^2+^ hyperabsorption in TRPV5 knockout mice. J. Am. Soc. Nephrol..

[154] Nijenhuis T., Van Der Eerden B.C., Hoenderop J.G., Weinans H., van Leeuwen J.P., Bindels R.J. (2008). Bone resorption inhibitor alendronate normalizes the reduced bone thickness of TRPV5(-/-) mice. J. Bone Miner. Res. Off. J. Am. Soc. Bone Miner. Res..

[155] Yelshanskaya M.V., Nadezhdin K.D., Kurnikova M.G., Sobolevsky A.I. (2021). Structure and function of the calcium-selective TRP channel TRPV6. J. Physiol..

[156] Bianco S.D., Peng J.B., Takanaga H., Suzuki Y., Crescenzi A., Kos C.H., Zhuang L., Freeman M.R., Gouveia C.H., Wu J. (2007). Marked disturbance of calcium homeostasis in mice with targeted disruption of the Trpv6 calcium channel gene. J. Bone Miner. Res. Off. J. Am. Soc. Bone Miner. Res..

[157] Yao D., Huang L., Ke J., Zhang M., Xiao Q., Zhu X. (2020). Bone metabolism regulation: Implications for the treatment of bone diseases. Biomed. Pharmacother..

[158] Srivastava R.K., Sapra L., Mishra P.K. (2022). Osteometabolism: Metabolic Alterations in Bone Pathologies. Cells.

[159] Gavva N.R., Treanor J.J., Garami A., Fang L., Surapaneni S., Akrami A., Alvarez F., Bak A., Darling M., Gore A. (2008). Pharmacological blockade of the vanilloid receptor TRPV1 elicits marked hyperthermia in humans. Pain.

[160] Shibata M., Tang C. (2021). Implications of Transient Receptor Potential Cation Channels in Migraine Pathophysiology. Neurosci. Bull..

[161] Fischer S.P.M., Brusco I., Brum E.S., Fialho M.F.P., Camponogara C., Scussel R., Machado-de-Ávila R.A., Trevisan G., Oliveira S.M. (2020). Involvement of TRPV1 and the efficacy of α-spinasterol on experimental fibromyalgia symptoms in mice. Neurochem. Int..

[162] Nahama A., Ramachandran R., Cisternas A.F., Ji H. (2020). The role of afferent pulmonary innervation in ARDS associated with COVID-19 and potential use of resiniferatoxin to improve prognosis: A review. Med. Drug Discov..

[163] Caterina M.J. (2007). Transient receptor potential ion channels as participants in thermosensation and thermoregulation. Am. J. Physiol. Regul. Integr. Comp. Physiol..

[164] Luo J., Hu H., Islas L.D., Qin F. (2014). Chapter Twelve—Thermally Activated TRPV3 Channels. Current Topics in Membranes.

[165] Yamada T., Ueda T., Ugawa S., Ishida Y., Imayasu M., Koyama S., Shimada S. (2010). Functional expression of transient receptor potential vanilloid 3 (TRPV3) in corneal epithelial cells: Involvement in thermosensation and wound healing. Exp. Eye Res..

[166] Randhawa P.K., Jaggi A.S. (2015). TRPV4 channels: Physiological and pathological role in cardiovascular system. Basic Res. Cardiol..

[167] Itoh Y., Hatano N., Hayashi H., Onozaki K., Miyazawa K., Muraki K. (2009). An environmental sensor, TRPV4 is a novel regulator of intracellular Ca2+ in human synoviocytes. Am. J. Physiol. Cell Physiol..

[168] Kang S.S., Shin S.H., Auh C.K., Chun J. (2012). Human skeletal dysplasia caused by a constitutive activated transient receptor potential vanilloid 4 (TRPV4) cation channel mutation. Exp. Mol. Med..

[169] Zhong G., Long H., Chen F., Yu Y. (2021). Oxoglaucine mediates Ca2+ influx and activates autophagy to alleviate osteoarthritis through the TRPV5/calmodulin/CAMK-II pathway. Br. J. Pharmacol..

[170] Lieben L., Benn B.S., Ajibade D., Stockmans I., Moermans K., Hediger M.A., Peng J.B., Christakos S., Bouillon R., Carmeliet G. (2010). Trpv6 mediates intestinal calcium absorption during calcium restriction and contributes to bone homeostasis. Bone.

[171] Ho K.W., Ward N.J., Calkins D.J. (2012). TRPV1: A stress response protein in the central nervous system. Am. J. Neurodegener. Dis..

[172] Cho W.G., Valtschanoff J.G. (2008). Vanilloid receptor TRPV1-positive sensory afferents in the mouse ankle and knee joints. Brain Res.

[173] Li B., Yang X.-Y., Qian F.-P., Tang M., Ma C., Chiang L.-Y. (2015). A novel analgesic approach to optogenetically and specifically inhibit pain transmission using TRPV1 promoter. Brain Res..

[174] Han P., Korepanova A.V., Vos M.H., Moreland R.B., Chiu M.L., Faltynek C.R. (2013). Quantification of TRPV1 protein levels in rat tissues to understand its physiological roles. J. Mol. Neurosci..

[175] Kochukov M.Y., Mcnearney T.A., Fu Y., Westlund K.N. (2006). Thermosensitive TRP ion channels mediate cytosolic calcium response in human synoviocytes. Am. J. Physiol. Cell Physiol..

[176] Valdes A.M., De Wilde G., Doherty S.A., Lories R.J., Vaughn F.L., Laslett L.L., Maciewicz R.A., Soni A., Hart D.J., Zhang W. (2011). The Ile585Val TRPV1 variant is involved in risk of painful knee osteoarthritis. Ann. Rheum. Dis..

[177] El Karim I., Mccrudden M.T., Linden G.J., Abdullah H., Curtis T.M., McGahon M., About I., Irwin C., Lundy F.T. (2015). TNF-α-induced p38MAPK activation regulates TRPA1 and TRPV4 activity in odontoblast-like cells. Am. J. Pathol..

[178] Spahn V., Stein C., Zöllner C. (2014). Modulation of transient receptor vanilloid 1 activity by transient receptor potential ankyrin 1. Mol. Pharmacol..

[179] Xiao T., Sun M., Kang J., Zhao C. (2022). Transient Receptor Potential Vanilloid1 (TRPV1) Channel Opens Sesame of T Cell Responses and T Cell-Mediated Inflammatory Diseases. Front. Immunol..

[180] Majhi R.K., Sahoo S.S., Yadav M., Pratheek B.M., Chattopadhyay S., Goswami C. (2015). Functional expression of TRPV channels in T cells and their implications in immune regulation. FEBS J..

[181] Lv Z., Xu X., Sun Z., Yang Y.X., Guo H., Li J., Sun K., Wu R., Xu J., Jiang Q. (2021). TRPV1 alleviates osteoarthritis by inhibiting M1 macrophage polarization via Ca(2+)/CaMKII/Nrf2 signaling pathway. Cell Death Dis..

[182] Engler A., Aeschlimann A., Simmen B.R., Michel B.A., Gay R.E., Gay S., Sprott H. (2007). Expression of transient receptor potential vanilloid 1 (TRPV1) in synovial fibroblasts from patients with osteoarthritis and rheumatoid arthritis. Biochem. Biophys. Res. Commun..

[183] Dewaker V., Sharma A.R., Debnath U., Park S.T., Kim H.S. (2023). Insights from molecular dynamics simulations of TRPV1 channel modulators in pain. Drug Discov. Today.

[184] Gouin O., L’herondelle K., Lebonvallet N., Le Gall-Ianotto C., Sakka M., Buhé V., Plée-Gautier E., Carré J.-L., Lefeuvre L., Misery L. (2017). TRPV1 and TRPA1 in cutaneous neurogenic and chronic inflammation: Pro-inflammatory response induced by their activation and their sensitization. Protein Cell.

[185] Takahashi N., Matsuda Y., Sato K., de Jong P.R., Bertin S., Tabeta K., Yamazaki K. (2016). Neuronal TRPV1 activation regulates alveolar bone resorption by suppressing osteoclastogenesis via CGRP. Sci. Rep..

[186] Nakamoto H., Katanosaka Y., Chijimatsu R., Mori D., Xuan F., Yano F., Omata Y., Maenohara Y., Murahashi Y., Kawaguchi K. (2021). Involvement of Transient Receptor Potential Vanilloid Channel 2 in the Induction of Lubricin and Suppression of Ectopic Endochondral Ossification in Mouse Articular Cartilage. Arthritis Rheumatol..

[187] Laragione T., Harris C., Gulko P.S. (2019). TRPV2 suppresses Rac1 and RhoA activation and invasion in rheumatoid arthritis fibroblast-like synoviocytes. Int. Immunopharmacol..

[188] Somogyi C.S., Matta C., Foldvari Z., Juhász T., Katona É., Takács Á R., Hajdú T., Dobrosi N., Gergely P., Zákány R. (2015). Polymodal Transient Receptor Potential Vanilloid (TRPV) Ion Channels in Chondrogenic Cells. Int. J. Mol. Sci..

[189] Halonen L., Pemmari A., Nummenmaa E., Hämäläinen M., Moilanen T., Vuolteenaho K., Moilanen E. (2023). Human Osteoarthritic Chondrocytes Express Nineteen Different TRP-Genes-TRPA1 and TRPM8 as Potential Drug Targets. Int. J. Mol. Sci..

[190] Yin S., Zhang L., Ding L., Huang Z., Xu B., Li X., Wang P., Mao J. (2018). Transient receptor potential ankyrin 1 (trpa1) mediates il-1β-induced apoptosis in rat chondrocytes via calcium overload and mitochondrial dysfunction. J. Inflamm..

[191] Willard V.P., Leddy H.A., Palmer D., Wu C.L., Liedtke W., Guilak F. (2021). Transient receptor potential vanilloid 4 as a regulator of induced pluripotent stem cell chondrogenesis. Stem Cells.

[192] Muramatsu S., Wakabayashi M., Ohno T., Amano K., Ooishi R., Sugahara T., Shiojiri S., Tashiro K., Suzuki Y., Nishimura R. (2007). Functional gene screening system identified TRPV4 as a regulator of chondrogenic differentiation. J. Biol. Chem..

[193] Clark A.L., Votta B.J., Kumar S., Liedtke W., Guilak F. (2010). Chondroprotective role of the osmotically sensitive ion channel transient receptor potential vanilloid 4: Age-and sex-dependent progression of osteoarthritis in Trpv4-deficient mice. Arthritis Rheum..

[194] Phan M.N., Leddy H.A., Votta B.J., Kumar S., Levy D.S., Lipshutz D.B., Lee S.H., Liedtke W., Guilak F. (2009). Functional characterization of TRPV4 as an osmotically sensitive ion channel in porcine articular chondrocytes. Arthritis Rheum..

[195] Masuyama R., Mizuno A., Komori H., Kajiya H., Uekawa A., Kitaura H., Okabe K., Ohyama K., Komori T. (2012). Calcium/calmodulin-signaling supports TRPV4 activation in osteoclasts and regulates bone mass. J. Bone Miner. Res. Off. J. Am. Soc. Bone Miner. Res..

[196] Pettenuzzo S., Arduino A., Belluzzi E., Pozzuoli A., Fontanella C.G., Ruggieri P., Salomoni V., Majorana C., Berardo A. (2023). Biomechanics of Chondrocytes and Chondrons in Healthy Conditions and Osteoarthritis: A Review of the Mechanical Characterisations at the Microscale. Biomedicines.

[197] Gao W., Hasan H., Anderson D.E., Lee W. (2022). The Role of Mechanically-Activated Ion Channels Piezo1, Piezo2, and TRPV4 in Chondrocyte Mechanotransduction and Mechano-Therapeutics for Osteoarthritis. Front. Cell Dev. Biol..

[198] Fu S., Meng H., Inamdar S., Das B., Gupta H., Wang W., Thompson C.L., Knight M.M. (2021). Activation of TRPV4 by mechanical, osmotic or pharmaceutical stimulation is anti-inflammatory blocking IL-1β mediated articular cartilage matrix destruction. Osteoarthr. Cartil..

[199] Khatib N.S., Monsen J., Ahmed S., Huang Y., Hoey D.A., Nowlan N.C. (2023). Mechanoregulatory role of TRPV4 in prenatal skeletal development. Sci. Adv..

[200] Mizoguchi F., Mizuno A., Hayata T., Nakashima K., Heller S., Ushida T., Sokabe M., Miyasaka N., Suzuki M., Ezura Y. (2008). Transient receptor potential vanilloid 4 deficiency suppresses unloading-induced bone loss. J. Cell. Physiol..

[201] O’conor C.J., Leddy H.A., Benefield H.C., Liedtke W.B., Guilak F. (2014). TRPV4-mediated mechanotransduction regulates the metabolic response of chondrocytes to dynamic loading. Proc. Natl. Acad. Sci. USA.

[202] Zhang M., Meng N., Wang X., Chen W., Zhang Q. (2022). TRPV4 and PIEZO Channels Mediate the Mechanosensing of Chondrocytes to the Biomechanical Microenvironment. Membranes.

[203] Guo D., Lin C., Lu Y., Guan H., Qi W., Zhang H., Shao Y., Zeng C., Zhang R., Zhang H. (2022). FABP4 secreted by M1-polarized macrophages promotes synovitis and angiogenesis to exacerbate rheumatoid arthritis. Bone Res..

[204] Sun H., Sun Z., Xu X., Lv Z., Li J., Wu R., Fei Y., Tan G., Liu Z., Liu Y. (2022). Blocking TRPV4 Ameliorates Osteoarthritis by Inhibiting M1 Macrophage Polarization via the ROS/NLRP3 Signaling Pathway. Antioxidants.

[205] Zhou X., Wang W., Miao J., Bai L. (2014). Expression and significance of transient receptor potential cation channel V5 in articular cartilage cells under exercise loads. Biomed. Rep..

[206] Chen R., Zhou X., Yin S., Lu Z., Nie J., Zhou W., Liu X. (2018). Study on the protective mechanism of autophagy on cartilage by magnesium sulfate. Chin. J. Reparative Reconstr. Surg..

[207] Hdud I.M., El-Shafei A.A., Loughna P., Barrett-Jolley R., Mobasheri A. (2012). Expression of Transient Receptor Potential Vanilloid (TRPV) channels in different passages of articular chondrocytes. Int. J. Mol. Sci..

[208] Wei Y., Zheng D., Guo X., Zhao M., Gao L., Bai L. (2018). Transient Receptor Potential Channel, Vanilloid 5, Induces Chondrocyte Apoptosis in a Rat Osteoarthritis Model through the Mediation of Ca^2+^ Influx. Cell. Physiol. Biochem..

[209] Wei Y., Jin Z., Zhang H., Piao S., Lu J., Bai L. (2018). The Transient Receptor Potential Channel, Vanilloid 5, Induces Chondrocyte Apoptosis via Ca2+ CaMKII-Dependent MAPK and Akt/ mTOR Pathways in a Rat Osteoarthritis Model. Cell. Physiol. Biochem..

[210] Song T., Ma J., Guo L., Yang P., Zhou X., Ye T. (2017). Regulation of chondrocyte functions by transient receptor potential cation channel V6 in osteoarthritis. J. Cell. Physiol..

[211] Haywood A.R., Hathway G.J., Chapman V. (2018). Differential contributions of peripheral and central mechanisms to pain in a rodent model of osteoarthritis. Sci. Rep..

[212] Rosenbaum T., Islas L.D. (2023). Molecular Physiology of TRPV Channels: Controversies and Future Challenges. Annu. Rev. Physiol..

[213] Yelshanskaya M.V., Sobolevsky A.I. (2022). Ligand-Binding Sites in Vanilloid-Subtype TRP Channels. Front. Pharmacol..

[214] Logashina Y.A., Palikova Y.A., Palikov V.A., Kazakov V.A., Smolskaya S.V., Dyachenko I.A., Tarasova N.V., Andreev Y.A. (2021). Anti-Inflammatory and Analgesic Effects of TRPV1 Polypeptide Modulator APHC3 in Models of Osteo- and Rheumatoid Arthritis. Mar. Drugs.

[215] Mlost J., Kostrzewa M., Malek N., Starowicz K. (2018). Molecular Understanding of the Activation of CB1 and Blockade of TRPV1 Receptors: Implications for Novel Treatment Strategies in Osteoarthritis. Int. J. Mol. Sci..

[216] Atobe M., Nagami T., Muramatsu S., Ohno T., Kitagawa M., Suzuki H., Ishiguro M., Watanabe A., Kawanishi M. (2019). Discovery of Novel Transient Receptor Potential Vanilloid 4 (TRPV4) Agonists as Regulators of Chondrogenic Differentiation: Identification of Quinazolin-4(3 H)-ones and in Vivo Studies on a Surgically Induced Rat Model of Osteoarthritis. J. Med. Chem..

[217] Xu B., Xing R., Huang Z., Yin S., Li X., Zhang L., Ding L., Wang P. (2019). Excessive mechanical stress induces chondrocyte apoptosis through TRPV4 in an anterior cruciate ligament-transected rat osteoarthritis model. Life Sci..

[218] Brown W., Leff R.L., Griffin A., Hossack S., Aubray R., Walker P., Chiche D.A. (2017). Safety, Pharmacokinetics, and Pharmacodynamics Study in Healthy Subjects of Oral NEO6860, a Modality Selective Transient Receptor Potential Vanilloid Subtype 1 Antagonist. J. Pain.

[219] Stevens R.M., Ervin J., Nezzer J., Nieves Y., Guedes K., Burges R., Hanson P.D., Campbell J.N. (2019). Randomized, Double-Blind, Placebo-Controlled Trial of Intraarticular Trans-Capsaicin for Pain Associated With Osteoarthritis of the Knee. Arthritis Rheumatol..

[220] Stevens R., Hanson P., Tiseo P., Guedes K., Campbell J., Connolly J., Ruggiero S., Corliss M., Smith V., Conaghan P.G. (2020). Op0187 determining optimal cooling and administration methods for cntx-4975 intra-articular injection in subjects with moderate to severe osteoarthritis knee pain. Ann. Rheum. Dis..

[221] Othman A.A., Nothaft W., Awni W.M., Dutta S. (2013). Effects of the TRPV1 antagonist ABT-102 on body temperature in healthy volunteers: Pharmacokinetic/ pharmacodynamic analysis of three phase 1 trials. Br. J. Clin. Pharmacol..

[222] Othman A.A., Nothaft W., Awni W.M., Dutta S. (2012). Pharmacokinetics of the TRPV1 antagonist ABT-102 in healthy human volunteers: Population analysis of data from 3 phase 1 trials. J. Clin. Pharmacol..

[223] Manitpisitkul P., Flores C.M., Moyer J.A., Romano G., Shalayda K., Tatikola K., Hutchison J.S., Mayorga A.J. (2018). A multiple-dose double-blind randomized study to evaluate the safety, pharmacokinetics, pharmacodynamics and analgesic efficacy of the TRPV1 antagonist JNJ-39439335 (mavatrep). Scand. J. Pain.

[224] Mayorga A.J., Flores C.M., Trudeau J.J., Moyer J.A., Shalayda K., Dale M., Frustaci M.E., Katz N., Manitpisitkul P., Treister R. (2017). A randomized study to evaluate the analgesic efficacy of a single dose of the TRPV1 antagonist mavatrep in patients with osteoarthritis. Scand. J. Pain.

[225] Iftinca M., Defaye M., Altier C. (2021). TRPV1-Targeted Drugs in Development for Human Pain Conditions. Drugs.

[226] Brown D.C. (2016). Resiniferatoxin: The Evolution of the “Molecular Scalpel” for Chronic Pain Relief. Pharmaceuticals.

[227] Zhang M., Ma Y., Ye X., Zhang N., Pan L., Wang B. (2023). TRP (transient receptor potential) ion channel family: Structures, biological functions and therapeutic interventions for diseases. Signal Transduct. Target. Ther..

[228] Miller F., Björnsson M., Svensson O., Karlsten R. (2014). Experiences with an adaptive design for a dose-finding study in patients with osteoarthritis. Contemp. Clin. Trials.

